# Nocturnal sleep duration and bone mineral density: a cross-sectional study of the National Health and Nutrition Examination Survey (NHANES) 2007–2014

**DOI:** 10.1186/s12902-022-01259-1

**Published:** 2022-12-28

**Authors:** Yuchen Tang, Jinmin Liu, Zhiwei Feng, Zhongcheng Liu, Shenghong Wang, Yayi Xia, Bin Geng

**Affiliations:** 1 Department of Orthopaedics, Lanzhou University Second Hospital, #82 Cuiyingmen, Gansu 730000 Lanzhou, China; 2 Orthopaedics Key Laboratory of Gansu Province, Lanzhou, Gansu China; 3 Orthopaedic Clinical Research Center of Gansu Province, Lanzhou, Gansu China

**Keywords:** Sleep duration, Bone mineral density, Vitamin D, Vitamin D intake, 25-hydroxyvitamin D

## Abstract

**Background:**

This study aimed to investigate the association between sleep duration and bone mineral density (BMD) and determine whether vitamin D (VD) status influenced the association between sleep duration and BMD.

**Methods:**

National Health and Nutrition Examination Survey 2007–2014 participants aged ≥ 40 years were included in this study. BMD testing was conducted with dual-energy X-ray absorptiometry examinations. Moreover, all individuals were divided into four groups according to self-reported nocturnal sleep duration (7–8 h; 6 h; < 6 h; and > 8 h). In addition, the differences in BMD between the normal sleep duration group and other groups were calculated using multiple linear regression models.

**Results:**

Overall, the median age of the overall study population was 55.00 years old, with 46.97% of men distributed. Participants sleeping > 8 h/night had lower BMDs than those sleeping 7–8 h/night. Moreover, the association between unhealthy sleep duration (especially > 8 h/night) and low BMD was more pronounced in older individuals, men, postmenopausal women, and subjects with inadequate VD intakes (< 15.00 µg/day) or deficient/insufficient serum 25-hydroxyvitamin D (< 75.00 nmol/L).

**Conclusions:**

In conclusion, unhealthy sleep duration, especially long sleep duration, was associated with decreased BMD, particularly among individuals aged > 60 years, men, or postmenopausal women. Moreover, VD status might influence the association between sleep duration and BMD, especially in the context of inadequate VD intake or deficient/insufficient serum 25-hydroxyvitamin D levels. However, given the limitations of the present study, further investigation is warranted to confirm this association and to explore potential mechanisms.

**Supplementary Information:**

The online version contains supplementary material available at 10.1186/s12902-022-01259-1.

## Background

Osteoporosis, which is characterized by low bone mineral density (BMD) and microarchitectural deterioration of bone tissue, is a common chronic disease among the middle-age and elderly individuals [1]. According to the International Osteoporosis Foundation (IOF), one-third of females and one-fifth of males aged over 50 years worldwide are at risk of osteoporosis, and the prevalence of osteoporosis is still increasing annually in the middle-age and elderly population [2–4]. Moreover, osteoporotic fracture, which is one of the most severe complications of osteoporosis, is an important cause of morbidity among older adults [5, 6]. In addition to placing an economic burden on healthcare systems, osteoporosis also drastically impacts the quality of life of patients [1]. Osteoporosis is a complex disease determined by numerous genes and environmental factors from the standpoint of pathogenesis [1]. In addition to essential nonmodifiable risk factors, such as aging, menopausal status, and genetic factors, many modifiable risk factors, such as dietary and lifestyle habits, also play essential roles in the pathogenesis of osteoporosis [1]. Therefore, exploration of the potentially modifiable risk factors for osteoporosis is receiving increasing attention and is expected to open new preventive avenues.

Although several previous studies reported that long or short sleep duration might be associated with lower BMD or a high risk of osteoporosis [7, 8]. Fu et al. observed that Chinese women who slept 6 h or less per night showed lower BMD than those who slept 8 h per night [7]. Wang et al. found that post-menopausal women with long sleep duration (> 10 h/day) showed a higher risk of osteoporosis than those with normal sleep duration (8–9 h/day) [8]. However, it should be noted that there is still no reliable conclusion because some studies found that long or short sleep durations might not contribute to decreased BMD [9, 10]. For example, Swanson et al. observed that nocturnal sleep duration was not independently associated with hip BMD among postmenopausal women irrespective of the method of assessment of sleep duration (objective or subjective) [9]. Another study by Swanson et al. found that there were no significant differences in hip or spine BMD between older men with short sleep duration (< 6 h) or recommended sleep duration (7–8 h) [10]. These controversial findings (abnormal sleep duration might be associated with low BMD or it might not) indicate that more studies on the impact of sleep duration on bone health are needed and suggest that other potential factors might influence the association between sleep duration and BMD.

As previous studies have reported, vitamin D (VD) is an essential nutrient for human health [11, 12]. VD status usually involves two aspects: daily VD intake and serum 25-hydroxyvitamin D [25(OH)D] level. VD intake from dietary sources and supplements is essential for maintaining adequate VD levels in the body, especially for individuals with insufficient light exposure. Moreover, the serum level of 25(OH)D, a precursor of activated VD, reflects whole-body VD stores and is the most commonly used indicator to adjudicate whether VD deficiency is present. Several studies have demonstrated that VD plays an essential role in bone metabolism [11, 12]. Low daily VD intake or low serum 25(OH)D levels were associated with lower BMD [11, 12]. Moreover, many studies found that vitamin status was linked to sleep behaviors [13–15]. For example, Majid et al. observed that VD supplements improved sleep quality among individuals aged 20 to 50 years [16]. A meta-analysis by Gao et al. found that VD deficiency was associated with poor sleep quality, short sleep duration, and sleepiness [15]. In addition, de Oliveira et al. observed that short sleep duration (< 6 h/day) was independently associated with low serum 25(OH)D levels among men [17]. However, there was no definite evidence of whether VD status affected the association between sleep duration and BMD.

Based on the above reasons, this study aimed to investigate the association between sleep duration and BMD. Moreover, we also tried to determine whether VD status affected the association between sleep duration and BMD.

## Methods

### Study population

This was a cross-sectional study. The data of all participants were extracted from the National Health and Nutrition Examination Survey (NHANES) database [18]. The NHANES database, which is affiliated with the Centers for Disease Control and Prevention (USA) and updated biennially, was designed to assess the health and nutritional status of general United States (US) residents. We extracted data from the NHANES database (2007–2008, 2009–2010, 2013–2014; BMD data were not obtainable in the NHANES database for 2011–2012) [18]. The inclusion criteria were as follows: (i) participants aged ≥ 40 years; (ii) participants with complete BMD data; (iii) participants with available sleep duration data; and (iv) participants with complete VD status data (VD intake and serum 25(OH)D). Moreover, subjects with missing data on covariates (missing data; refused to answer; or answered “do not know”) were excluded from the present study. Finally, all individuals included in this study provided informed consent, and the ethics review board of the National Center for Health Statistics approved the study [19].

### BMD testing

All participants included in the present study underwent BMD testing via dual-energy X-ray absorptiometry (DXA) examinations. The examinations were conducted by certified radiology technologists using Hologic QDR-4500A fan-beam densitometers (Hologic; Bedford, MA), and the data analysis was performed using Hologic APEX, version 4.0, software. Other details about the procedure of BMD testing are available on the NHANES website [20]. Moreover, this study analyzed the BMD data of the femoral regions [total femur BMD (TF-BMD) and femoral neck BMD (FN-BMD)] and spinal areas [total spine BMD (TS-BMD)].

### Sleep duration

The sleep duration analyzed in the present study included only self-reported nocturnal sleep duration. Moreover, the present study did not include daytime sleep duration or daytime napping because they were not available on the NHANES database. All sleep duration data were collected by a questionnaire survey, which asked the participants, “How much sleep do you usually get at night on weekdays or workdays?” Moreover, all results collected for sleep duration were reported in integral numbers. Other details about the sleep duration data collection are available on the NHANES website [21]. Moreover, we divided all participants into four groups according to the frequency distribution of sleep duration among all individuals included in the final analysis (Supplementary Figure S1) and NIH sleep duration recommendations (7–8 h/day) [22]: (i) Group 1 (normal sleep duration, *N* = 2371): 7 to 8 h/day; (ii) Group 2 (*N* = 1106): 6 h/day; (3) Group 3 (*N* = 644): < 6 h/day; and (4) Group 4 (*N* = 278): > 8 h/day.

### Vitamin D status

VD status included two aspects in this study: VD intake levels and serum 25(OH)D levels. For VD intake (D2 + D3), NHANES assessed the types and amounts of foods/beverages (including all types of water) consumed and dietary supplements used during the 24-h period prior to the interview and estimated the intakes of VD from those foods/beverages and dietary supplements. Information on VD intake was collected through in-person interviews and telephone surveys (3 to 10 days after the in-person interview). The dietary recall statuses were classified as (i) reliable and met the minimum criteria; (ii) not reliable or did not meet the minimum criteria; (iii) reported consuming breast milk (for infants); and (iv) not done. In the present study, we enrolled only participants with a dietary recall status that was “reliable and met the minimum criteria” in the final analysis. Moreover, to balance both methods (in person or by phone) of errors, we calculated the mean values between the two methods to investigate VD intake and used them as the final values of VD intake. Other details about the measurement of VD intake are available on the NHANES website [23]. Finally, according to the published Endocrine Society’s Practice Guidelines on Vitamin D [24], individuals were divided into two groups according to VD intake levels: (i) adequate VD intake: VD intake ≥ 15.00 μg/day; and (ii) inadequate VD intake: VD intake < 15.00 μg/day.

Serum 25(OH)D levels (D2 + D3) were detected in the serum of participants. Moreover, the quantitative detection of 25-hydroxyvitamin concentration was performed by ultra-high-performance liquid chromatography-tandem mass spectrometry. The lower limits of detection (LODs) for 25(OH)D2 and 25(OH)D3 were 2.05 nmol/L and 2.23 nmol/L, respectively. Serum 25(OH)D concentrations less than the LOD were imputed as LOD/√2. Other details about the detection of the serum 25(OH)D concentrations are available on the NHANES website [25]. Finally, the participants with different serum 25(OH)D levels were grouped into two categories according to the published Endocrine Society’s Practice Guidelines on Vitamin D [24]: (i) deficient/insufficient serum 25(OH)D levels: serum 25(OH)D < 75.00 nmol/L; (ii) sufficient serum 25(OH)D levels: serum 25(OH)D ≥ 75.00 nmol/L [individuals with ≥ 125 nmol/L serum 25(OH)D, which was considered to have potential toxic effects on human health [26], were also assigned to sufficient serum 25(OH)D levels group because the sample size was too small (*N* = 110)].

### Covariates

Considering the impact of other factors on BMD, we controlled for potential variables to perform covariate-adjusted analyses. The selection of potential variables that might affect BMD was performed according to previous studies and accessibility data from the NHANES database [1, 27]. Finally, age, sex/menopause status (the definition and information extraction of menopause status referred to a previous study [28]), race, education level, income level, body mass index (BMI), smoking status, alcohol drinking status, physical activity level, sedentary activity, fracture history, glucocorticoid use, family history of osteoporosis, Charlson Comorbidity Index (CCI) (the definition and information extraction of CCI referred to a previous study [29]), calcium intake and caffeine intake were considered to be potential covariates in the present study. Other detailed information on the covariates is listed in Supplementary Table S1.

### Statistical analysis

First, all analyses were based on participants with complete data; thus, the individuals with missing data on covariates were excluded from the final analysis. Second, the baseline characteristics were indicated by the mean/median (continuous variables) and proportion (categorical variable). Moreover, the normality of the continuous variables was tested with the Shapiro–Wilk normality test. If the data of the continuous variable were normally distributed, statistical significance was determined using Student’s t-test; if the data were nonnormally distributed, statistical significance was determined using the Kruskal–Wallis test. For categorical variables, statistical significance was determined using the chi-square test. Third, covariates associated with TF-BMD with a p-value of < 0.05 in univariate analysis were included in the multiple linear regression models. Finally, the differences in BMD between participants with normal sleep duration and any other group were also assessed using multiple linear regression models. All analyses were performed using R software (version 4.0.3; https://www.R-project.org) and EmpowerStats (version 2.0; http://www.empowerstats.com). *P* values < 0.05 were considered statistically significant.

## Results

### Participant selection and baseline characteristics

**Fig. 1:**
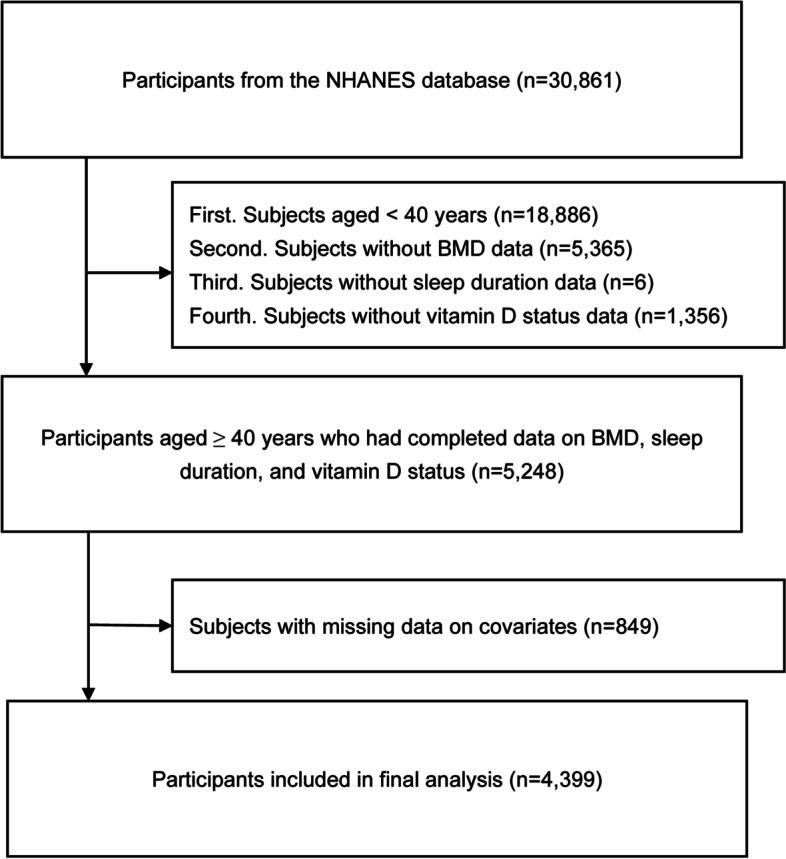
Flowchart of participants selection. NHANES, National Health and Nutrition Examination Survey; BMD, bone mineral density

The flow chart of participant selection is displayed in Fig. 1. We extracted the information of 30,861 participants from the NHANES 2007–2014. First, we excluded subjects aged < 40 years of age (*n* = 18,886), subjects without complete BMD data (*n* = 5,365), subjects without SD data (*n* = 6), and subjects without VD status data (*n* = 1,356). Second, we excluded individuals with missing data on covariates (details on the missing covariate data are shown in Supplementary Fig. S2; *n* = 849), and a total of 4,399 participants were included in the final analysis.

**Fig. 2:**
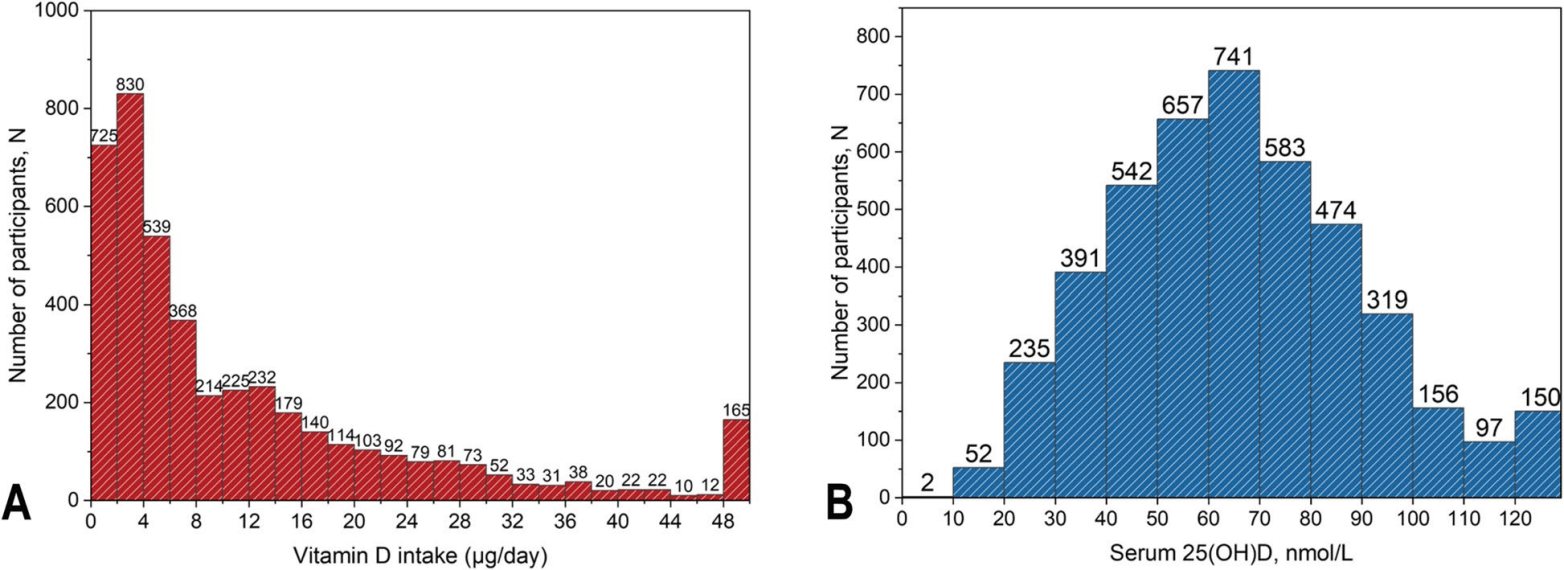
Frequency distributions for vitamin D intake and serum 25(OH)D. **A** Vitamin D; **B** Serum 25(OH)D. 25(OH)D, 25-hydroxyvitamin D

The baseline characteristics of the individuals are listed in Table 1. Overall, the median age of the overall study population was 55.00 years old, with 46.97% of men and 53.03% of women distributed. Compared with individuals with 7–8 h of sleep per day, individuals with > 8 h of sleep per day showed lower TF-BMD and FN-BMD, while individuals with < 6 or > 8 h of sleep per day showed higher TF-BMD and FN-BMD than those with 7–8 h of sleep per day. Moreover, individuals sleeping < 6 h/ day showed lower vitamin D intakes than those sleeping 7–8 h/day. In addition, individuals with < 6 or 6 h of sleep per day showed lower serum 25(OH)D levels than those with 7–8 h of sleep per day. To make the results more intuitive, we also plotted the frequency histogram of the number of individuals with different VD intake and serum 25(OH)D. The frequency histogram is shown in Fig. 2.

**Table 1:** Characteristics of participants included in final analysis

Characteristics	Group	Total	Group 1 (SD = 7–8 h/day)	Group 2 (SD = 6 h/day)	Group 3 (SD < 6 h/day)	Group 4 (SD > 8 h/day)
		***N* = 4399**	***N* = 2371**	***N* = 1106**	***N* = 644**	***N* = 278**
Age (year)^a^		55.00 [46.00,65.00]	55.00 [46.00,65.00]	54.00 [46.00,63.00] ^*^	53.00 [46.00,62.00] ^**^	60.00 [47.25,72.75] ^**^
Sex/menopause status n, (%)	Men	2066 (46.97%)	1097 (46.27)	547 (49.46)	302 (46.89)	120 (43.17)
	Non-postmenopausal women	816 (18.55%)	444 (18.73)	202 (18.26)	114 (17.70)	56 (20.14)
	Postmenopausal women	1517 (34.49%)	830 (35.01)	357 (32.28)	228 (35.40)	102 (36.69)
Race n, (%)	Mexican American	711 (16.16%)	399 (16.83)	181 (16.37) ^**^	94 (14.60) ^**^	37 (13.31)
	Other Hispanic	439 (9.98%)	220 (9.28)	121 (10.94) ^**^	81 (12.58) ^**^	17 (6.12)
	Non-Hispanic White	2186 (49.69%)	1290 (54.41)	496 (44.85) ^**^	232 (36.02) ^**^	168 (60.43)
	Non-Hispanic Black	785 (17.84%)	324 (13.67)	223 (20.16) ^**^	194 (30.12) ^**^	44 (15.83)
	Other Race	278 (6.32%)	138 (5.82)	85 (7.69) ^**^	43 (6.68) ^**^	12 (4.32)
Education level n, (%)	Under high school	1083 (24.62%)	551 (23.24)	252 (22.78)	195 (30.28) ^**^	85 (30.58) ^**^
	High school or equivalent	995 (22.62%)	502 (21.17)	269 (24.32)	157 (24.38) ^**^	67 (24.10) ^**^
	Above high school	2321 (52.76%)	1318 (55.59)	585 (52.89)	292 (45.34) ^**^	126 (45.32) ^**^
Income level n, (%)	Q1 (PIR: 0.00–1.23)	1082 (24.60%)	533 (22.48)	267 (24.14) ^**^	206 (31.99) ^**^	76 (27.34) ^**^
	Q2 (PIR: 1.24–2.47)	1117 (25.39%)	617 (26.02)	249 (22.51) ^**^	172 (26.71) ^**^	79 (28.42) ^**^
	Q3 (PIR: 2.48–4.63)	1093 (24.85%)	553 (23.32)	305 (27.58) ^**^	161 (25.00) ^**^	74 (26.62) ^**^
	Q4 (PIR: 4.64–5.00)	1107 (25.16%)	668 (28.17)	285 (25.77) ^**^	105 (16.30) ^**^	49 (17.63) ^**^
BMI n, (%)	Normal (BMI < 25 kg/m2)	1220 (27.73%)	691 (29.14)	293 (26.49) ^*^	154 (23.91) ^**^	82 (29.50)
	Overweight (30 > BMI ≥ 25 kg/m2)	1656 (37.64%)	929 (39.18)	409 (36.98) ^*^	216 (33.54) ^**^	102 (36.69)
	Obese (BMI ≥ 30 kg/m2)	1523 (34.62%)	751 (31.67)	404 (36.53) ^*^	274 (42.55) ^**^	94 (33.81)
Smoking status n, (%)	Currently smoking	795 (18.07%)	375 (15.82)	216 (19.53) ^*^	152 (23.60) ^**^	52 (18.71)
	Ex-smoking	1237 (28.12%)	686 (28.93)	291 (26.31) ^*^	178 (27.64) ^**^	82 (29.50)
	Never smoke	2367 (53.81%)	1310 (55.25)	599 (54.16) ^*^	314 (48.76) ^**^	144 (51.80)
Alcohol drinking status n, (%)	Currently drinking	2890 (65.70%)	1605 (67.69)	730 (66.00)	399 (61.96) ^**^	156 (56.12) ^**^
	Ex-drinking	924 (21.00%)	453 (19.11)	226 (20.43)	165 (25.62) ^**^	80 (28.78) ^**^
	Never drink	585 (13.30%)	313 (13.20)	150 (13.56)	80 (12.42) ^**^	42 (15.11) ^**^
Physical activity level n, (%)	NMVPA (0 MET-mins/week)	1154 (26.23%)	582 (24.55)	288 (26.04)	180 (27.95)	104 (37.41) ^**^
	LMVPA (1–599 MET-mins/week)	629 (14.30%)	346 (14.59)	156 (14.10)	88 (13.66)	39 (14.03) ^**^
	MMVPA (600–1199 MET-mins/week)	526 (11.96%)	306 (12.91)	119 (10.76)	68 (10.56)	33 (11.87) ^**^
	HMVPA (≥ 1200 MET-mins/week)	2090 (47.51%)	1137 (47.95)	543 (49.10)	308 (47.83)	102 (36.69) ^**^
Sedentary activity n, (%)	Q1 (0–150 min/day)	786 (17.87%)	396 (16.70)	196 (17.72)	159 (24.69) ^**^	35 (12.59)
	Q2 (151–240 min/day)	1118 (25.41%)	611 (25.77)	255 (23.06)	175 (27.17) ^**^	77 (27.70)
	Q3 (241–479 min/day)	1118 (25.41%)	632 (26.66)	284 (25.68)	135 (20.96) ^**^	67 (24.10)
	Q4 (≥ 480 min/day)	1377 (31.30%)	732 (30.87)	371 (33.54)	175 (27.17) ^**^	99 (35.61)
Fracture history n, (%)	No	3921 (89.13%)	2130 (89.84)	991 (89.60)	564 (87.58)	236 (84.89) ^*^
	Yes	478 (10.87%)	241 (10.16)	115 (10.40)	80 (12.42)	42 (15.11) ^*^
Glucocorticoid use n, (%)	Yes	256 (5.82%)	114 (4.81)	71 (6.42)	48 (7.45) ^*^	23 (8.27) ^*^
	No	4143 (94.18%)	2257 (95.19)	1035 (93.58)	596 (92.55) ^*^	255 (91.73) ^*^
Family history of osteoporosis n, (%)	Yes	625 (14.21%)	333 (14.04)	160 (14.47)	95 (14.75)	37 (13.31)
	No	3774 (85.79%)	2038 (85.96)	946 (85.53)	549 (85.25)	241 (86.69)
CCI	G1: CCI = 0	2015 (45.81%)	1142 (48.17)	502 (45.39)	271 (42.08) ^*^	100 (35.97) ^**^
	G2: CCI = 1	1028 (23.37%)	538 (22.69)	260 (23.51)	153 (23.76) ^*^	77 (27.70) ^**^
	G3: CCI > 1	1356 (30.83%)	691 (29.14)	344 (31.10)	220 (34.16) ^*^	101 (36.33) ^**^
Calcium intake n, (%)	Q1 (39.50–655.50 mg/day)	1100 (25.01%)	547 (23.07)	287 (25.95)	196 (30.43) ^**^	70 (25.18)
	Q2: (655.51–965.00 mg/day)	1098 (24.96%)	599 (25.26)	262 (23.69)	172 (26.71) ^**^	65 (23.38)
	Q3: (965.01–1398.00 mg/day)	1101 (25.03%)	584 (24.63)	284 (25.68)	156 (24.22) ^**^	77 (27.70)
	Q4 (> 1398.00 mg/day)	1100 (25.01%)	641 (27.04)	273 (24.68)	120 (18.63) ^**^	66 (23.74)
Caffeine intake n, (%)	Q1 (0.00–39.00 mg/day)	1099 (24.98%)	576 (24.29)	266 (24.05)	173 (26.86)	84 (30.22)
	Q2 (39.01–115.00 mg/day)	1098 (24.96%)	602 (25.39)	286 (25.86)	144 (22.36)	66 (23.74)
	Q3 (115.01–224.50 mg/day)	1101 (25.03%)	578 (24.38)	290 (26.22)	162 (25.16)	71 (25.54)
	Q4 (> 224.50 mg/day)	1101 (25.03%)	615 (25.94)	264 (23.87)	165 (25.62)	57 (20.50)
Vitamin D _(D2+D3)_ intake (μg/day)^a^		6.45 [2.80,15.85]	7.25 [3.00,17.02]	6.18 [2.76,15.24]	4.90 [2.20,11.70] ^**^	7.12 [3.30,15.49]
Serum 25(OH)D _(D2 + D3)_ (nmol/L)^a^		64.20 [48.00,81.80]	66.10 [50.60,83.70]	63.50 [47.90,81.45] ^**^	58.45 [39.70,74.93] ^**^	63.85 [47.42,80.68]
TF-BMD (g/cm2)^a^		0.95 [0.84,1.05]	0.94 [0.84,1.05]	0.96 [0.85,1.07] ^*^	0.96 [0.86,1.07] ^*^	0.91 [0.80,1.03] ^**^
FN-BMD (g/cm2)^a^		0.79 [0.70,0.89]	0.79 [0.70,0.88]	0.80 [0.71,0.90] ^**^	0.81 [0.71,0.90] ^**^	0.76 [0.66,0.86] ^**^
TS-BMD (g/cm2)^a^		1.01 [0.91,1.12]	1.01 [0.91,1.11]	1.02 [0.91,1.13]	1.01 [0.90,1.12]	1.00 [0.89,1.10]

*BMD* bone mineral density, *BMI* body mass index, *CCI* Charlson Comorbidity Index, *FN* femoral neck, *HMVPA* high moderate-to-vigorous physical activity, *LMVPA* low moderate-to-vigorous physical activity, *MMVPA* medium moderate-to-vigorous physical activity, *NMVPA* no moderate-to-vigorous physical activity, *PIR* poverty income ratio, *SD* sleep duration, *TF* total femur, *TS* total spine, *25(OH)D* 25-hydroxyvitamin D

^a ^Not normally distributed variables (by Shapiro–Wilk normality test)

^*^ *p* < 0.05 compared with Group1

^**^ *p* < 0.01 compared with Group 1

### Selection of covariates

We included fifteen candidate covariates in the univariate analysis and then selected covariates with a *p*-value of < 0.05 for association with TF-BMD. The results of the univariate analysis are presented in Supplementary Table S2. Finally, we included age, sex/menopause status, race, income level, BMI, smoking status, alcohol drinking status, physical activity level, fracture history, glucocorticoid use, family history of osteoporosis, CCI, calcium intake in the multiple linear regression models.

### Association between sleep duration and BMD

**Fig. 3:**
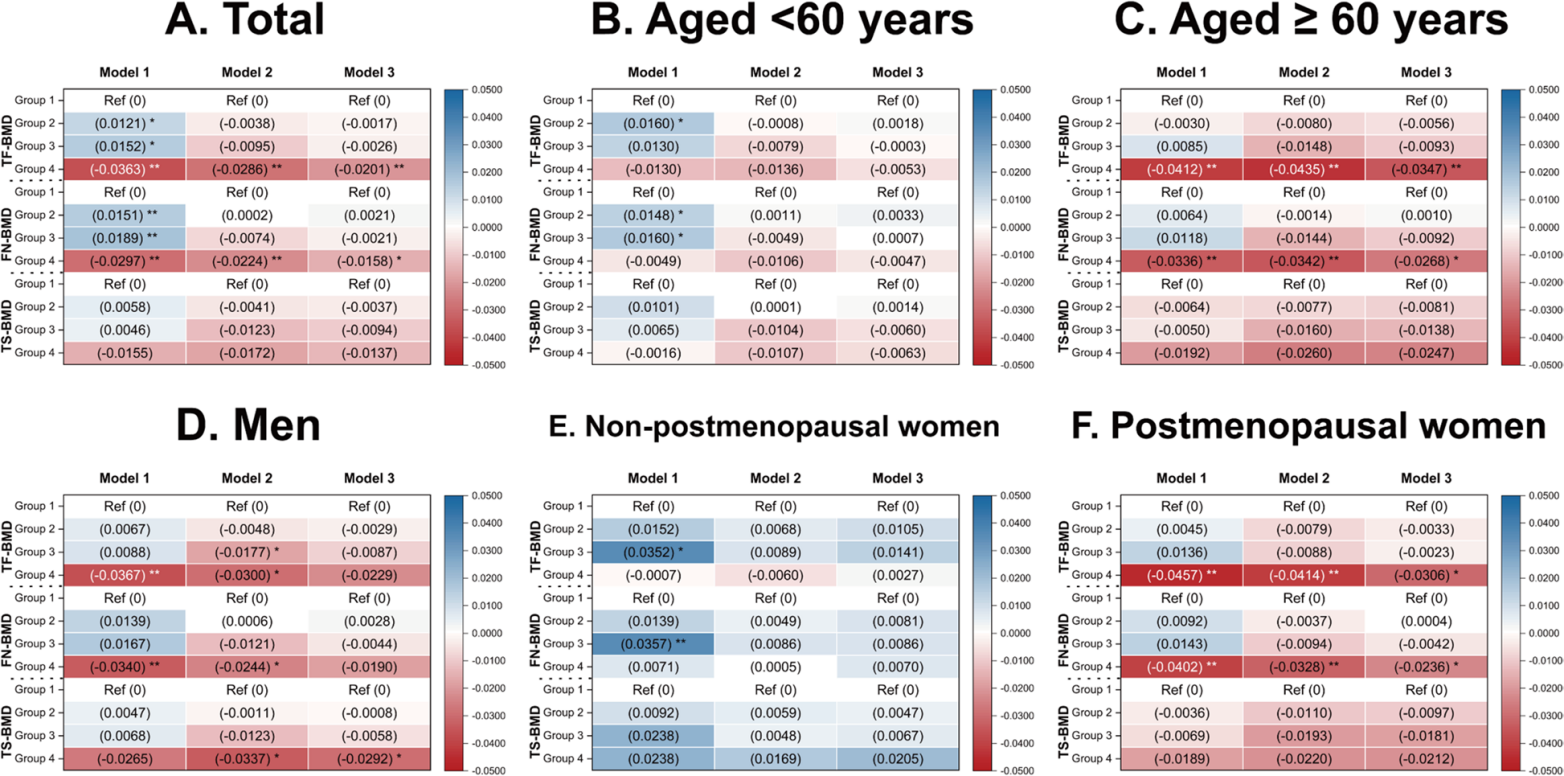
Association between sleep duration and BMD. **A** overall population; **B** individuals aged < 60 years; **C** individuals aged ≥ 60 years; **D** men; **E** non-postmenopausal women; **F** postmenopausal women. Model 1: unadjusted model; Model 2: age, sex/menopause status, race, and BMI were adjusted; Model 3: age, sex/menopause status, race, income level, BMI, smoking status, alcohol drinking status, physical activity level, fractures history, glucocorticoid use, family history of osteoporosis, CCI, calcium intake, vitamin D intake, and serum 25-hydroxyvitamin D were adjusted. Group 1: SD = 7–8 h/day; Group 2: SD = 6 h/day; Group3: SD < 6 h/day; Group 4: SD > 8 h/day. BMD, bone mineral density; BMI, body mass index; CCI, Charlson Comorbidity Index; FN, femoral neck; SD, sleep duration; TF, total femur; TS, total spine. * *p* < 0.05; ** *p* < 0.01

Overall, individuals with > 8 h of sleep per day showed lower TF-BMD and FN-BMD than those with 7–8 h of sleep per day, while higher TF-BMD and FN-BMD were observed among individuals sleeping six or < 6 h/day in Model 1 (unadjusted model). Moreover, when age, sex/menopause status, race, and BMI were adjusted (Model 2), significant differences in TF-BMD and FN-BMD between individuals sleeping > 8 h/day and 7–8 h/day were observed. In addition, when all covariates, VD intakes, and serum 25(OH)D levels were adjusted (Model 3), the results suggested that participants with > 8 h of sleep per day had significantly lower TF-BMD and FN-BMD than those with 7–8 h of sleep per day. The specific results are listed in Fig. 3, Supplementary Tables S3 and S4.

When stratified by age, individuals (aged < 60 years) sleeping six h/night showed higher TF-BMD and FN-BMD, and individuals sleeping < 6 h/night showed higher  FN-BMD than those with 7–8 h of sleep per day in Model 1. However, no significant differences in BMDs were observed in any abnormal sleep duration group (< 6, 6, or > 8 h/day) compared with the normal sleep duration group (7–8 h/day) in Model 2 and Model 3. Moreover, individuals (aged ≥ 60 years) sleeping > 8 h/day showed lower TF-BMD and FN-BMD than those sleeping 7–8 h/day, regardless of adjustment. The details are listed in Fig. 3, Supplementary Tables S3, and S4.

When stratified by sex/menopause status, men sleeping > 8 h/day showed lower TF-BMD and FN-BMD than those with 7–8 h of sleep per day in Model 1. Moreover, men sleeping > 8 h/day still showed lower TF-BMD, FN-BMD, and TS-BMD, and men sleeping < 6 h/day showed lower TF-BMD compared with those sleeping 7–8 h/day in Model 2. In addition, men sleeping > 8 h/night still showed lower TS-BMD than those sleeping 7–8 h/day in Model 3. Non-postmenopausal women sleeping < 6 h/day showed higher TF-BMD and FN-BMD than those sleeping 7–8 h/night in Model 1. Moreover, postmenopausal women sleeping > 8 h/day showed significantly lower TF-BMD and FN-BMD than those sleeping 7–8 h/day, regardless of adjustment. The details are listed in Fig. 3, Supplementary Tables S3 and S4.

### Association between sleep duration and BMD in different vitamin D intake levels

**Fig. 4:**
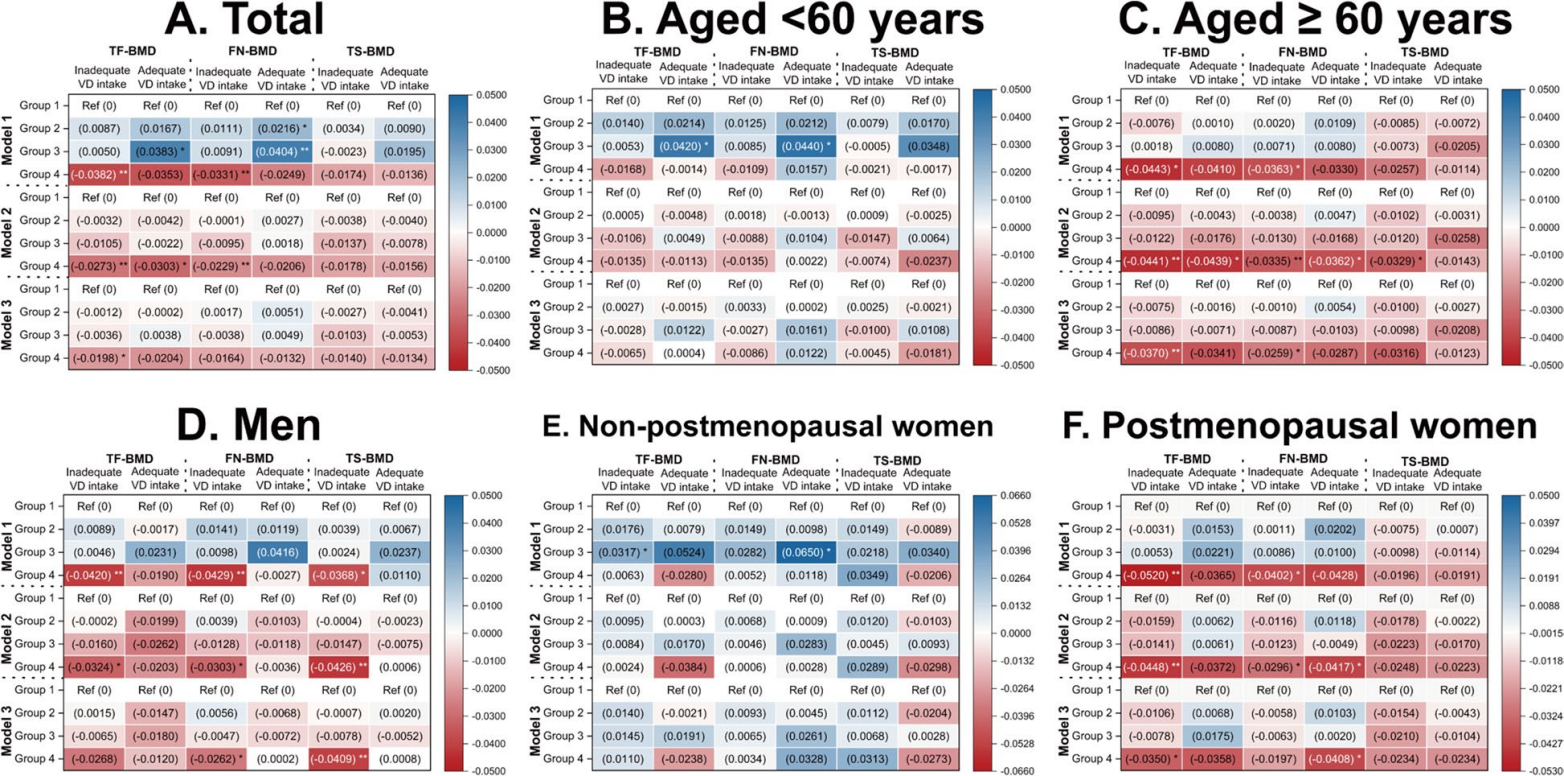
Association between sleep duration and BMD in different vitamin D intake levels. **A** overall population; **B** individuals aged < 60 years; **C** individuals aged ≥ 60 years; **D** men; **E** non-postmenopausal women; **F** postmenopausal women. Model 1: unadjusted model; Model 2: age, sex/menopause status, race, and BMI were adjusted; Model 3: age, sex/menopause status, race, income level, BMI, smoking status, alcohol drinking status, physical activity level, fractures history, glucocorticoid use, family history of osteoporosis, CCI, calcium intake, and serum 25-hydroxyvitamin D were adjusted. Group 1: SD = 7–8 h/day; Group 2: SD = 6 h/day; Group3: SD < 6 h/day; Group 4: SD > 8 h/day. BMD, bone mineral density; BMI, body mass index; CCI, Charlson Comorbidity Index; FN, femoral neck; SD, sleep duration; TF, total femur; TS, total spine. * *p* < 0.05; ** *p* < 0.01

Overall, in the inadequate VD intake group (< 15.00 µg/day), individuals sleeping > 8 h/day showed lower TF-BMD and FN-BMD than those sleeping 7–8 h/day in Model 1 (unadjusted model) and Model 2 (age, sex/ menopause status, race, and BMI were adjusted). Moreover, individuals sleeping > 8 h/night showed lower TF-BMD than those sleeping 7–8 h/day in Model 3 [all covariates and serum 25(OH)D levels were adjusted]. In the adequate VD intake group (≥ 15.00 µg/day), individuals sleeping six h/night showed higher FN-BMD, and individuals sleeping < 6 h/day showed higher TF-BMD and FN-BMD than those sleeping 7–8 h/day in Model 1. Moreover, individuals sleeping > 8 h/day showed lower TF-BMD than those sleeping 7–8 h/day in Model 2. However, no significant differences in BMDs were observed in any abnormal sleep duration group (< 6, 6, or > 8 h/day) compared with the normal sleep duration group (7–8 h/day) in Model 3. The details are listed in Fig. 4, Supplementary Tables S3 and S5.

When stratified by age, among subjects with inadequate VD intake, individuals (aged ≥ 60 years) sleeping > 8 h/day showed significantly lower TF-BMD (Model 1–3), FN-BMD (Model 1–3), and TS-BMD (Model 2) than those sleeping 7–8 h/day. In the adequate VD intake group, individuals (aged < 60 years) sleeping < 6 h/ day showed higher TF-BMD and FN-BMD than the control group in Model 1. Moreover, individuals (aged ≥ 60 years) sleeping > 8 h/day showed lower TF-BMD and FN-BMD than those sleeping 7–8 h/night in Model 2. The details are listed in Fig. 4, Supplementary Tables S3 and S5.

When stratified by sex/menopause status, in the inadequate VD intake group, non-postmenopausal women sleeping < 6 h/day showed higher TF-BMD (Model 1) than those sleeping 7–8 h/day. Moreover, men sleeping > 8 h/day showed significantly lower TF-BMD (Model 1–2), FN-BMD (Model 1–3), and TS-BMD (Model 1–3) than those sleeping 7–8 h/day. In addition, postmenopausal women sleeping > 8 h/day showed significantly lower TF-BMD (Model 1–3) and FN-BMD (Model 1–2) than those sleeping 7–8 h/day. In the adequate VD intake group, non-postmenopausal women sleeping < 6 h/day showed higher FN-BMD (Model 1) than those sleeping 7–8 h/day. Moreover, postmenopausal women sleeping > 8 h/day showed lower FN-BMD (Model 2–3) than those sleeping 7–8 h/day. The details are listed in Fig. 4, Supplementary Tables S3 and S5.

### Association between sleep duration and BMD in different serum 25(OH) levels

**Fig. 5:**
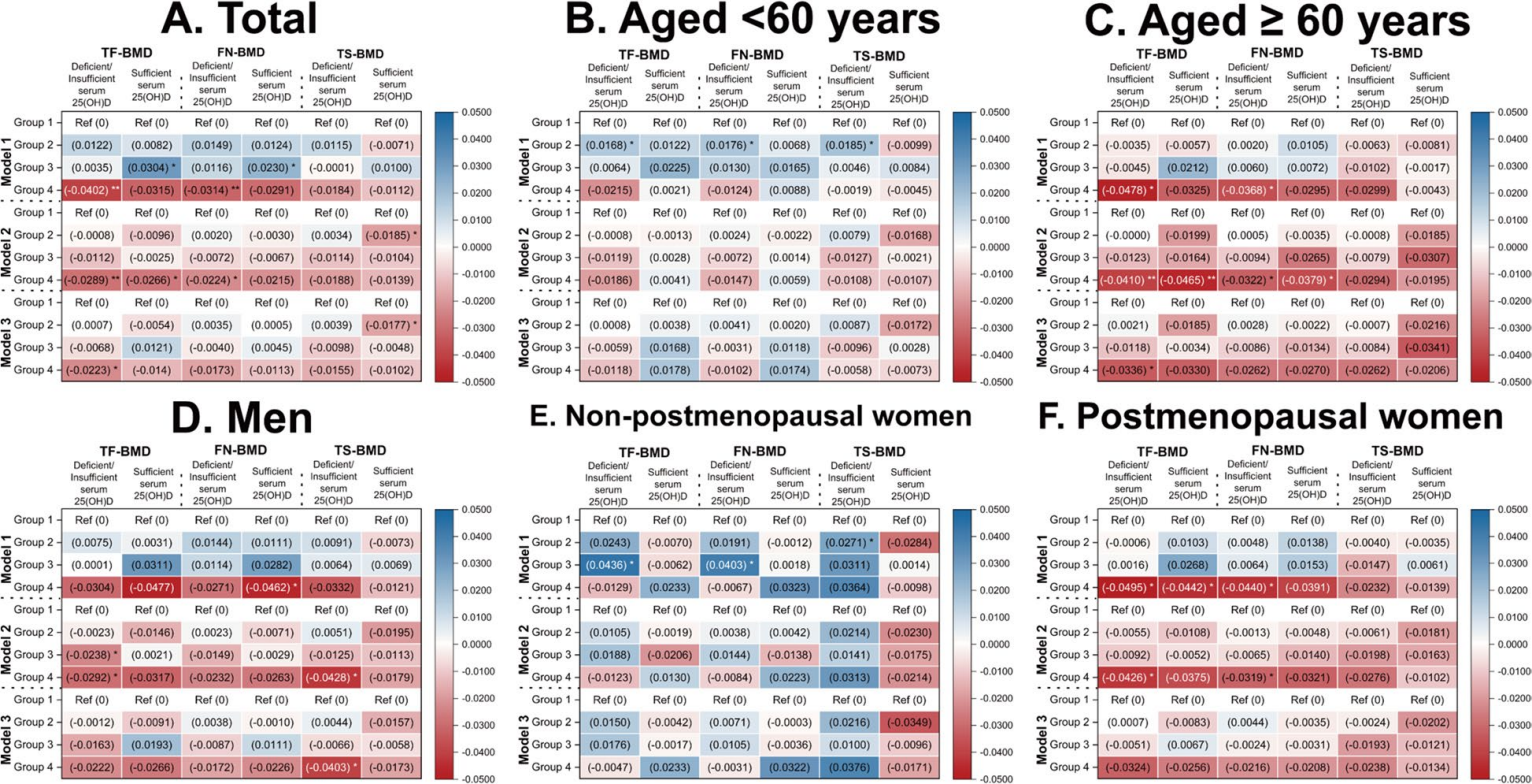
Association between sleep duration and BMD in different serum 25(OH) levels. **A** overall population; **B** individuals aged < 60 years; **C** individuals aged ≥ 60 years; **D** men; **E** non-postmenopausal women; **F** postmenopausal women. Model 1: unadjusted model; Model 2: age, sex/menopause status, race, and BMI were adjusted; Model 3: age, sex/menopause status, race, income level, BMI, smoking status, alcohol drinking status, physical activity level, fractures history, glucocorticoid use, family history of osteoporosis, CCI, calcium intake, and vitamin D intake were adjusted. Group 1: SD = 7–8 h/day; Group 2: SD = 6 h/day; Group3: SD < 6 h/day; Group 4: SD > 8 h/day. BMD, bone mineral density; BMI, body mass index; CCI, Charlson Comorbidity Index; FN, femoral neck; SD, sleep duration; TF, total femur; TS, total spine; 25(OH)D, 25-hydroxyvitamin D. * *p* < 0.05; ** *p* < 0.01

Overall, in the deficient/insufficient serum 25(OH)D group (0.00 ≤ serum 25(OH)D < 75.00 nmol/L), individuals with > 8 h of sleep per day showed lower TF-BMD and FN-BMD than those with 7–8 h of sleep per night in Model 1 (unadjusted model) and Model 2 ((age, sex/menopause, race, and BMI were adjusted). Moreover, individuals sleeping > 8 h/day showed lower TF-BMD than those sleeping 7–8 h/day in Model 3 (all covariates and VD intakes were adjusted). In the sufficient serum 25(OH)D group (serum 25(OH)D ≥ 75.00 nmol/L), individuals sleeping < 6 h/day showed higher TF-BMD and FN-BMD than those sleeping 7–8 h/day in Model 1. Moreover, individuals sleeping > 8 h/day showed lower TF-BMD than those sleeping 7–8 h/day in Model 2. In addition, individuals sleeping six h/day showed lower TS-BMD than those sleeping 7–8 h/day in Model 2 and Model 3. The details are listed in Fig. 5, Supplementary Tables S3 and S6.

When stratified by age, in the deficient/insufficient serum 25(OH)D group, individuals (aged < 60 years) sleeping six h/night had higher TF-BMD, FN-BMD, and TS-BMD than the control group (7–8 h/night) in Model 1. Moreover, individuals (aged ≥ 60 years) sleeping > 8 h/night showed lower TF-BMD (Model 1–3) and FN-BMD (Model 1–2) than the control group. In the sufficient serum 25(OH)D group, individuals (aged ≥ 60 years) sleeping > 8 h/night showed lower TF-BMD and FN-BMD than those sleeping 7–8 h/night (Model 2), while no significant differences were observed among individuals aged < 60 years. The details are listed in Fig. 5, Supplementary Tables S3 and S6.

When stratified by sex, in the deficient/insufficient serum 25(OH)D group, men sleeping < 6 h/day showed lower TF-BMD (Model 2), and men sleeping > 8 h/day showed significantly lower TF-BMD (Model 2) and TS-BMD (Model 2–3) than those sleeping 7–8 h/day. Moreover, non-postmenopausal women sleeping < 6 h/day showed higher TF-BMD and FN-BMD, and non-postmenopausal women sleeping six h/day showed higher TS-BMD than those sleeping 7–8 h/day in Model 1. In addition, postmenopausal women sleeping > 8 h/day showed significantly lower TF-BMD and FN-BMD than those sleeping 7–8 h/day in Model 2 and Model 3. In the sufficient serum 25(OH)D group, men sleeping > 8 h/night showed lower FN-BMD than the control group in Model 1, and postmenopausal women sleeping > 8 h/day showed significantly lower TF-BMD than those sleeping 7–8 h/day in Model 1. The details are listed in Fig. 5, Supplementary Tables S3 and S6.

## Discussion

Osteoporosis in middle-aged and older individuals has become a global issue in the past decade. Currently, there is an increasing awareness that dietary changes and lifestyle modification might be effective means to prevent osteoporosis. Our study found that unhealthy sleep duration (especially > 8 h/night) was associated with reduced BMD. Moreover, the negative effect of unhealthy sleep duration on BMD was more salient among older individuals (aged ≥ 60 years), men, postmenopausal women, and subjects with inadequate VD intake or deficient/insufficient serum 25(OH)D levels.

Some previous studies demonstrated that unhealthy sleep duration was associated with decreased BMD or increased risk of osteoporosis [9, 10, 30–32]. For example, Ochs-Balcom et al. observed that women with short sleep duration (≤ 5 h/per night) showed lower BMD and higher risk of low bone mass and osteoporosis than individuals with a 7-h sleep duration [30]. Tian et al. found that participants with a long sleep duration (≥ 9 h) had a higher risk of osteoporosis than those with sleep durations of 7–8 h regardless of sex [31]. A meta-analysis conducted by Wang et al. demonstrated that each one-hour reduction in sleep duration increased the risk of osteoporosis 1.03-fold, while a one-hour increment in sleep duration increased the risk of osteoporosis 1.01-fold [32]. The negative effect of unhealthy sleep duration on bone metabolism might involve the following mechanisms. First, unhealthy sleep duration might lead to reduced bone mass by affecting mechanical stress. As previously reported, mechanical stress plays an important role in maintaining bone mass [33, 34]. Moreover, mechanical stress is reduced during sleep, and long sleep duration might decrease the effect of mechanical stress on bone formation, which might be a possible explanation for the negative effect of long sleep duration on reduced bone mass. Second, unhealthy sleep duration might lead to reduced bone mass by affecting hormone levels in the body. For example, estrogen is considered the chief regulator of the balance between bone formation and resorption [35]. Michels et al. found that a one-hour increase in sleep duration was associated with a 0.039-fold increase in mean estradiol concentrations among women [36]. However, more studies to investigate whether unhealthy sleep duration leads to low BMD by affecting estrogen levels are needed because the number of related studies is limited. Moreover, melatonin has been shown to have an inhibitory effect on bone resorption and a promoting effect on bone formation. which received increasing attention in recent years because it is one of the most important factors in circadian regulation and plays a role in bone metabolism [37, 38]. Wu et al. observed that women with sleep durations ≥ 9 h showed 42% higher melatonin levels than those with sleep durations ≤ 6 h [39]. These results suggested that unhealthy sleep duration might influence BMD by affecting melatonin levels.

VD, a pro-hormone, is considered to play an essential role in bone metabolism [11, 12, 40]. On the one hand, VD could facilitate the intestinal absorption of calcium, which is an important metal ion in bone formation [11, 12, 40]. On the other hand, VD levels are closely related to parathyroid hormone (PTH) levels [12, 40]. PTH plays a crucial role in bone metabolism and calcium homeostasis. PTH is a major stimulator of VD synthesis in the kidney, and the levels of PTH are also regulated in a negative-feedback manner by VD [40]. Moreover, several studies demonstrated that there existed an intrinsic circadian rhythm of PTH secretion (two peaks: early morning and late evening) [41–44], which was affected by age and menopause status [41]. In addition, sleep duration might influence the rhythm of PTH and lead to a change in PTH levels. For example, Staab et al. observed a non-significant, but numerically, reduction in PTH levels caused by a short-term sleep restriction [45]. Therefore, long or short sleep duration might lead to decreased BMD by affecting the rhythm or levels of PTH, which is also a possible reason for the observed results that the negative effect of unhealthy sleep duration on BMD was more salient among individuals with inadequate VD status. Unfortunately, the information on PTH levels was not obtainable from the NHANES database. Therefore, we could not evaluate the level of PTH of the study population in the present study. However, this point mentioned above might be a topic worth pursuing in the future. Serum 25(OH)D, a precursor of activated VD, was considered an ideal indicator to reflect the storage of VD in the body [11, 12]. In the present study, we observed that the negative effects of unhealthy sleep duration on BMD were more obvious among individuals with inadequate VD intake or deficient/insufficient serum 25(OH)D levels, which suggested that VD status might influence the association between sleep duration and BMD or that sleep duration affects BMD by influencing VD levels in the body. In addition to bone metabolism, VD has also been associated with sleep, which is a complex concept including sleep duration and sleep quality, bedtime, and other aspects. A meta-analysis by Gao et al. found that VD deficiency was associated with poor sleep quality and sleepiness but not short sleep duration [15]. Similarly, Cheng et al. observed that women with plasma 25(OH)D deficiency showed a high risk of poor sleep quality [46]. Likewise, sleep behaviors might also affect the VD status. For example, de Oliveira et al. observed that short sleep duration (< 6 h/day) was independently associated with low serum 25(OH)D levels among men [17]. Moreover, some studies demonstrated that other factors involved in sleep were also associated with the risk of bone loss or osteoporosis. For example, Tang et al. found that some unhealthy sleep patterns (defined by sleep duration and bedtime) were associated with low femur BMD and a high risk of osteoporosis [47]. Similarly, Tian et al. found that individuals with long sleep duration (≥ 9 h) and early bedtime (sleep before 21:00 h) showed a high risk of osteoporosis [31]. In addition, Bevilacqua et al. found that self-reported sleep quality was associated with BMD in older adults [48]. Therefore, reduced BMD might be caused by low VD intake or low serum 25(OH)D levels, which affect sleep duration or other sleep-related behaviors or parameters. In comparison, sleep duration might affect BMD by influencing VD levels in the body, which might also be a plausible explanation for the negative effects of unhealthy sleep duration, especially long sleep duration, on BMD. Adequate VD intake is obviously important, but sun exposure also plays an essential role in maintaining adequate levels of activated VD [49]. On the one hand, long sleep duration might reduce the time of sun exposure, thus leading to VD deficiency. On the other hand, we speculate that unhealthy sleep duration might diminish the ability of the skin to produce VD, but this postulation needs further examination. Conversely, we also observed that postmenopausal women sleeping > 8 h/night showed lower FN-BMD than the control group (7–8 h/night) in those with adequate VD intake, which suggested that the complexity of the interaction between VD status and sleep duration. However, no significant differences were observed among female individuals with sufficient serum 25(OH)D levels. Moreover, the published Endocrine Society’s Practice Guidelines on Vitamin D also recommended that raising the blood level of 25(OH)D above 75 nmol/L may require at least 1500–2000 IU/d (37.5–50.0 µg/day) of supplemental VD [24]. These findings above suggested that 15 µg/day of VD intake might be inadequate, especially for women. In addition, because of the cross-sectional design of the present study, the causality requires further investigation.

In the present study, we observed that the adverse effects of abnormal sleep duration on BMD were more pronounced in individuals aged ≥ 60 years. On the one hand, it was consistently shown that aging was an important factor in bone loss [1], and individuals who were older appeared to have lower BMDs than those who were younger. On the other hand, aging was also associated with reduced hormone levels, especially sex hormones [50, 51], and older subjects seemed to have worse adaptability to the changes in metabolic or hormone levels caused by other factors. Moreover, we observed that the association between VD status and the negative effect of unhealthy sleep duration on BMD was different between men and women. On the one hand, the sex differences might result from the small sample size of the included population. On the other hand, the sex differences might suggest differences in VD requirements to maintain normal bone metabolism for men and women. The leading cause of bone loss among women is natural menopause [52]. Estrogen levels gradually decrease in older women after menopause, which disturbs the balance of bone metabolism and leads to diminished bone mass [52]. In comparison, the most important reason for bone loss or osteoporosis among men is aging, and the rate of disease progression among men was slower than that among women [1, 53]. Therefore, the theory presented above might explain the sex differences. Interestingly, we also observed differences in affected sites (femur or spine). Although there was no clear evidence to explain the differences in affected sites, several reasonable explanations can be put forth to account for this observation. For example, the biomechanical features of the femur and spine are different. Although the mechanical stimulus or stress on the femur and spine is lower during sleep, the mechanical stimulus or stress on the femur and spine generated by different physical activities might be different, which might be a reason for the differences in affected sites. For example, Hind et al. found that participants who participated in > 2 weeks of resistance training had significantly higher lumbar spine but not hip BMD than those who participated in < 2 weeks of resistance training [54]. However, considering that the type of physical activity could not be assessed among the individuals included, the differences in affected sites might result from the small sample size. Therefore, more studies will be needed to determine whether differences in affected sites exist and the detailed mechanism.

The findings of the present study might provide some valuable suggestions for clinical practice. First, our study revealed that unhealthy sleep duration might contribute to reduced bone mass. Therefore, maintaining a healthy sleep duration might be an effective approach to prevent bone loss. Moreover, this study revealed that the negative effect of unhealthy sleep duration on BMD might be affected by VD status, especially among individuals with inadequate VD intake or deficient/insufficient serum 25(OH)D levels. Therefore, adequate VD intake to maintain sufficient serum 25(OH)D levels might be a potential means to prevent the adverse effects of abnormal sleep duration on BMD. However, given the limitations of the present study (such as the cross-sectional study design), the causality between sleep duration and BMD and the specific role played by VD still need further investigation.

This study also had some limitations. First, only individuals with complete data were included, and subjects with missing data were excluded from the present study, which might produce bias. Second, sleep duration and dietary data were collected based on self-reports, which might not accurately reflect the actual situation and introduce recall bias. Third, the participants included in the final analysis were based on the general US population. Considering the differences in culture, lifestyle, and diet among different countries and regions, more studies are needed to investigate whether the conclusion of the present study is generally applicable. Fourth, the VD intake and sleep duration data were collected based on short-term intake, using short-term dietary intake as usual intake to assess whether VD intakes influenced the association between sleep duration and BMD, which might lead to a biased estimate. Fifth, some unmeasured confounding variables (such as medication use, PTH levels, bone turnover markers, sleep onset, and daylight hours), which are also considered important factors for bone metabolism and sleep behaviors, were not assessed in the present study because these variables were not available in the NHANES database, and the lack of adjustment for these potential factors may bias the results. Finally, the causality between sleep duration and BMD could not be established because this study was represented by a cross-sectional design.

## Conclusions

In conclusion, unhealthy sleep duration, especially long sleep duration, was associated with decreased BMD, particularly among individuals aged > 60 years, men, or postmenopausal women. Moreover, VD status might influence the association between sleep duration and BMD, especially in the context of inadequate VD intake or deficient/insufficient serum 25(OH)D levels. However, given the limitations of the present study, further investigation is warranted to confirm this association and to explore potential mechanisms.

## Supplementary Information

**Additional file 1: Supplementary Figure S1.** Frequency distribution of sleep duration among all individuals included in the final analysis. **Supplementary Figure S2.** Frequency distribution of sleep duration among all individuals included in the final analysis. **Supplementary  Table S1.** Detailed information on covariates. **Supplementary Table S2.** Univariate analysis for the association between covariates and total femur BMD. **Supplementary Table S3.** Number of participants in each subgroup. **Supplementary Table S4.** Association between sleep duration and BMD. **Supplementary Table S5.** Association between sleep duration and BMD in different vitamin D intake levels. **Supplementary Table S6.** Association between sleep duration and BMD in different serum 25(OH) levels.

## Data Availability

The datasets obtained and analysed during the current study are available on the NHANES database [https://www.cdc.gov/nchs/nhanes/index.htm].
