# Supplementary material for: Nocturnal sleep duration and bone mineral density: a cross-sectional study of the National Health and Nutrition Examination Survey (NHANES) 2007–2014
Source: BMC Endocr Disord. 2022 Dec 28;22:333. doi: 10.1186/s12902-022-01259-1 (PMC9795756; doi:10.1186/s12902-022-01259-1)
Supplement: Supplementary file 1 — Additional file 1: Supplementary Figure S1. Frequency distribution of sleep duration among all individuals included in the final analysis. Supplementary Figure S2. Frequency distribution of sleep duration among all individuals included in the final analysis. Supplementary Table S1. Detailed information on covariates. Supplementary Table S2. Univariate analysis for the association between covariates and total femur BMD. Supplementary Table S3. Number of participants in each subgroup. Supplementary Table S4. Association between sleep duration and BMD. Supplementary Table S5. Association between sleep duration and BMD in different vitamin D intake levels. Supplementary Table S6. Association between sleep duration and BMD in different serum 25(OH) levels. [file 12902_2022_1259_MOESM1_ESM.docx]

Supplementary Materials

**Supplementary Figure S1.** Frequency distribution of sleep duration among all individuals included in the final analysis

**Supplementary Figure S2.** Frequency distribution of sleep duration among all individuals included in the final analysis

**Supplementary Table S1.** Detailed information on covariates

**Supplementary Table S2.** Univariate analysis for the association between covariates and total femur BMD

**Supplementary Table S3.** Number of participants in each subgroup

**Supplementary Table S4.** Association between sleep duration and BMD

**Supplementary Table S5.** Association between sleep duration and BMD in different vitamin D intake levels

**Supplementary Table S6.** Association between sleep duration and BMD in different serum 25(OH) levels

**
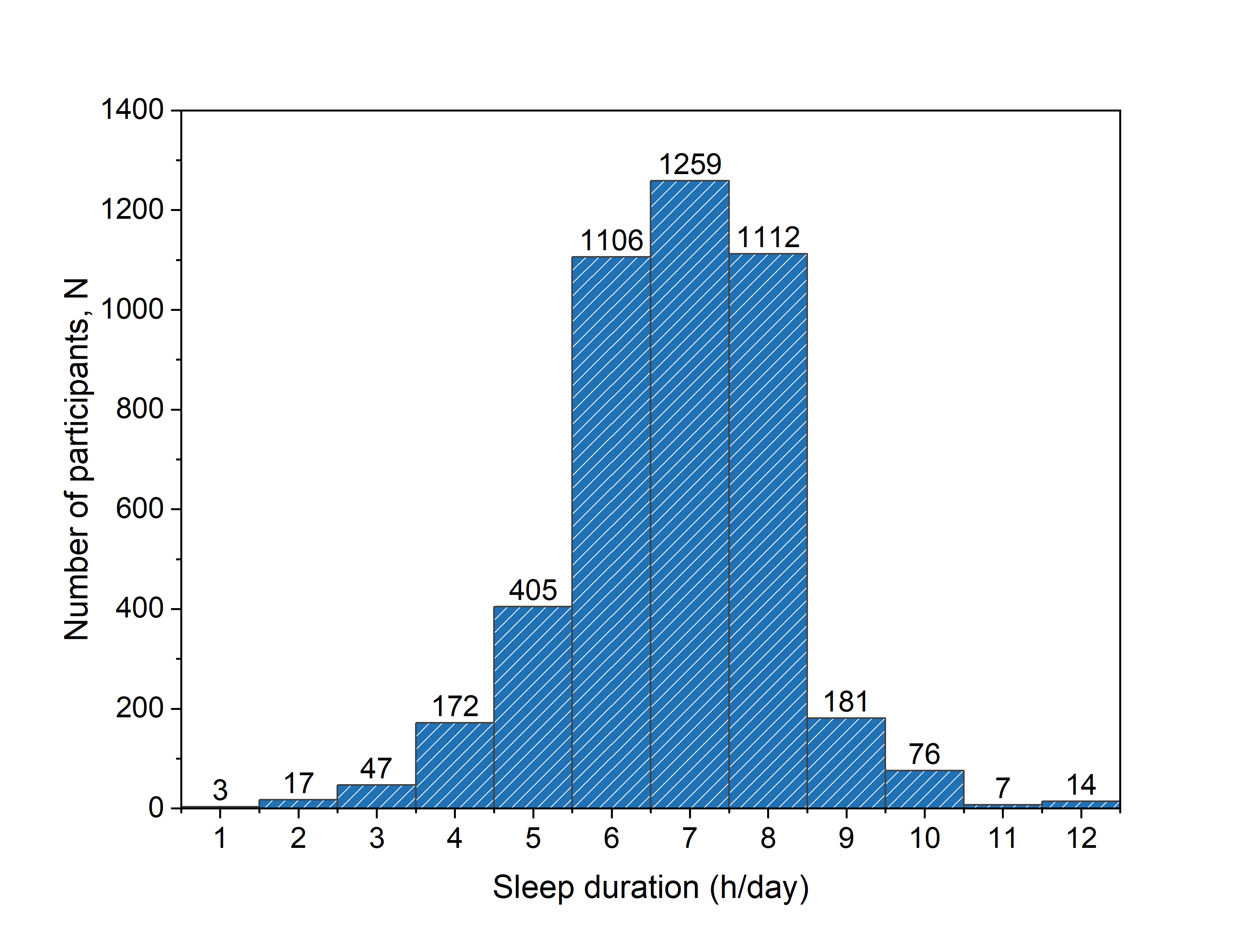
**

**Supplementary Figure S1** Frequency distribution of sleep duration among all individuals included in the final analysis.

**
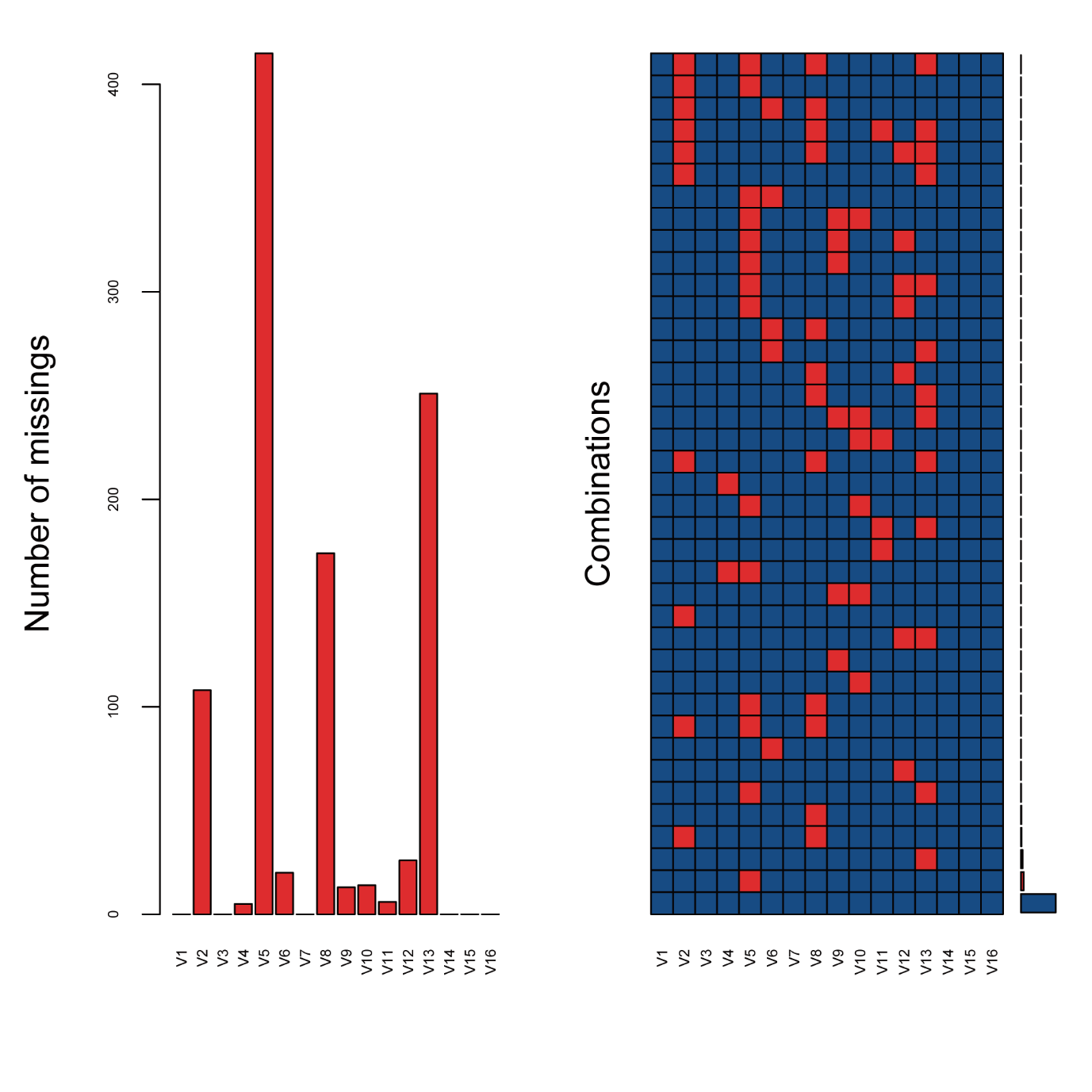
**

**Supplementary Figure S2** Missing data on covariates. V1, age; V2, sex and menopause status; V3, race; V4, education level; V5, income level; V6, BMI; V7, smoking status; V8, alcohol drinking status; V9, physical activity level; V10, sedentary activity; V11, fracture history; V12, glucocorticoid use; V13, family history of osteoporosis; V14, CCI; V15, calcium intake; V16, caffeine intake. BMI, body, mass index; CCI, Charlson Comorbidity Index.

**Supplementary Table S1** Detailed information on covariates

| **Covariate** | **Range (if continues variable)** | **Grouping for adjustment** |
| --- | --- | --- |
| **Age** | 40-80 years ("80" means ≥ 80 years of age) | 40-59 years |
|  |  | ≥ 60 years |
| **Sex/menopause status** | NA | Men |
|  |  | Non-postmenopausal women |
|  |  | Postmenopausal women |
| **Race** | NA | Mexican American |
|  |  | Other Hispanic |
|  |  | Non-Hispanic White |
|  |  | Non-Hispanic Black |
|  |  | Other Race - Including Multi-Racial |
| **Education level** | NA | Under high school |
|  |  | High school or equivalent |
|  |  | Above high school |
| **Income level** | PIR: 0.00-5.00 ("5.00" means ≥ 5.00) | Q1 (PIR: 0.00-1.23) |
|  |  | Q2 (PIR: 1.24-2.47) |
|  |  | Q3 (PIR: 2.48-4.63) |
|  |  | Q4 (PIR: 4.64-5.00) |
| **BMI** | 13.18-65.50 kg/m2 | Normal (BMI < 25kg/m^2^) |
|  |  | Overweight (25 ≤ BMI < 30kg/m^2^) |
|  |  | Obese (BMI ≥ 30kg/m^2^) |
| **Smoking status** | NA | Currently smoking |
|  |  | Ex-smoking |
|  |  | Never smoke |
| **Alcohol consumption** | NA | Currently drinking |
|  |  | Ex-drinking |
|  |  | Never drink |
| **Physical activity level** | 0-55440 MET-mins/week | HMVPA (≥ 1200 MET-mins/week) |
|  |  | MMVPA (600–1199 MET-mins/week) |
|  |  | LMVPA (1–599 MET-mins/week) |
|  |  | NMVPA (0 MET-mins/week) |
| **Sedentary activity** | 0-1200 mins/day | Q1 (0-150 mins/day) |
|  |  | Q2 (151-240 mins/day) |
|  |  | Q3 (241-479 mins/day) |
|  |  | Q4 (≥480 mins/day) |
| **Fracture history** | NA | Yes (self-report) |
|  |  | No |
| **Glucocorticoid use** | NA | Yes (Had glucocorticoid use) |
|  |  | No |
| **Family history of osteoporosis** | NA | Yes (patients with osteoporosis diagnosed by doctors) |
|  |  | No |
| **Charlson Comorbidity Index** | 0-12 | G1: CCI = 0 |
|  |  | G2: CCI = 1 |
|  |  | G3: CCI > 1 |
| **Calcium intake** | 39.50-5089.00 mg/day | Q1 (39.50-655.50 mg/day) |
|  |  | Q2: (655.51-965.00 mg/day) |
|  |  | Q3: (965.01-1398.00 mg/day) |
|  |  | Q4 (>1398.00 mg/day) |
| **Caffeine intake** | 0-2715.5 mg/day | Q1 (0.00-39.00 mg/day) |
|  |  | Q2 (39.01-115.00 mg/day) |
|  |  | Q3 (115.01-224.50 mg/day) |
|  |  | Q4 (>224.50 mg/day) |

BMI, body mass index; HMVPA, high moderate-to-vigorous physical activity; PIR, poverty income ratio; LMVPA, low moderate-to-vigorous physical activity; MMVPA, medium moderate-to-vigorous physical activity; NA, not applicable; NMVPA, no moderate-to-vigorous physical activity.

**Supplementary Table S2** Univariate analysis for the association between covariates and total femur BMD

| **Covariates** | **Group** | **N (%)** | **Total femur BMD** |
| --- | --- | --- | --- |
|  |  |  | **β (95% CI) P-value** |
| Age | < 60 years | 2692 (61.20%) | Reference |
|  | ≥ 60 years | 1707 (38.80%) | -0.078 (-0.087, -0.069) <0.001 |
| Sex and menopause status | Men | 2066 (46.97%) | Reference |
|  | Non-postmenopausal women | 816 (18.55%) | -0.053 (-0.065, -0.042) <0.001 |
|  | Postmenopausal women | 1517 (34.49%) | -0.156 (-0.165, -0.147) <0.001 |
| Race | Mexican American | 711 (16.16%) | Reference |
|  | Other Hispanic | 439 (9.98%) | -0.021 (-0.040, -0.003) 0.021 |
|  | Non-Hispanic White | 2186 (49.69%) | -0.032 (-0.045, -0.019) <0.001 |
|  | Non-Hispanic Black | 785 (17.84%) | 0.051 (0.036, 0.067) <0.001 |
|  | Other Race | 278 (6.32%) | -0.036 (-0.057, -0.015) <0.001 |
| Education level | Under high school | 1083 (24.62%) | Reference |
|  | High school or equivalent | 995 (22.62%) | 0.006 (-0.007, 0.020) 0.344 |
|  | Above high school | 2321 (52.76%) | 0.007 (-0.004, 0.018) 0.237 |
| Income level | Q1 (PIR: 0.00-1.23) | 1082 (24.60%) | Reference |
|  | Q2 (PIR: 1.24-2.47) | 1117 (25.39%) | 0.009 (-0.004, 0.022) 0.158 |
|  | Q3 (PIR: 2.48-4.63) | 1093 (24.85%) | 0.023 (0.009, 0.036) <0.001 |
|  | Q4 (PIR: 4.64-5.00) | 1107 (25.16%) | 0.031 (0.018, 0.044) <0.001 |
| BMI | Normal (BMI < 25kg/m2) | 1220 (27.73%) | Reference |
|  | Overweight (30 > BMI ≥ 25kg/m2) | 1656 (37.64%) | 0.093 (0.083, 0.104) <0.001 |
|  | Obese (BMI ≥ 30kg/m2) | 1523 (34.62%) | 0.150 (0.139, 0.161) <0.001 |
| Smoking status n, (%) | Currently smoking | 795 (18.07%) | Reference |
|  | Ex-smoking | 1237 (28.12%) | 0.024 (0.010, 0.038) <0.001 |
|  | Never smoke | 2367 (53.81%) | 0.021 (0.008, 0.033) 0.001 |
| Alcohol consumption n, (%) | Currently drinking | 2890 (65.70%) | Reference |
|  | Ex-drinking | 924 (21.00%) | -0.029 (-0.040, -0.017) <0.001 |
|  | Never drink | 585 (13.30%) | -0.052 (-0.066, -0.038) <0.001 |
| Physical activity level n, (%) | NMVPA (0 MET-mins/week) | 1154 (26.23%) | Reference |
|  | LMVPA (1–599 MET-mins/week) | 629 (14.30%) | 0.002 (-0.014, 0.017) 0.840 |
|  | MMVPA (600–1199 MET-mins/week) | 526 (11.96%) | 0.013 (-0.003, 0.029) 0.122 |
|  | HMVPA (≥ 1200 MET-mins/week) | 2090 (47.51%) | 0.041 (0.029, 0.052) <0.001 |
| Sedentary activity n, (%) | Q1 (0-150 mins/day) | 786 (17.87%) | Reference |
|  | Q2 (151-240 mins/day) | 1118 (25.41%) | -0.000 (-0.014, 0.014) 0.991 |
|  | Q3 (241-479 mins/day) | 1118 (25.41%) | -0.012 (-0.026, 0.002) 0.093 |
|  | Q4 (≥480 mins/day) | 1377 (31.30%) | 0.003 (-0.011, 0.016) 0.691 |
| Fracture history n, (%) | No | 3921 (89.13%) | Reference |
|  | Yes | 478 (10.87%) | -0.042 (-0.057, -0.027) <0.001 |
| Glucocorticoid use | Yes | 256 (5.82%) | Reference |
|  | No | 4143 (94.18%) | 0.047 (0.027, 0.067) <0.001 |
| Family history of osteoporosis | Yes | 625 (14.21%) | Reference |
|  | No | 3774 (85.79%) | 0.043 (0.030, 0.056) <0.001 |
| CCI | G1: CCI = 0 | 2015 (45.81%) | Reference |
|  | G2: CCI = 1 | 1028 (23.37%) | -0.022 (-0.034, -0.011) <0.001 |
|  | G3: CCI > 1 | 1356 (30.83%) | -0.029 (-0.040, -0.018) <0.001 |
| Calcium intake | Q1 (39.50-579.50 mg/day) | 1100 (25.01%) | Reference |
|  | Q2: (580.00-817.00 mg/day) | 1098 (24.96%) | 0.015 (0.002, 0.028) 0.027 |
|  | Q3: (817.50-1112.50 mg/day) | 1101 (25.03%) | 0.018 (0.005, 0.031) 0.007 |
|  | Q4 (1114.00-5046.50 mg/day) | 1100 (25.01%) | -0.010 (-0.023, 0.003) 0.132 |
| Caffeine intake | Q1 (0.00-39.50 mg/day) | 1099 (24.98%) | Reference |
|  | Q2 (40.00-115.50 mg/day) | 1098 (24.96%) | 0.002 (-0.011, 0.015) 0.797 |
|  | Q3 (116.00-224.50 mg/day) | 1101 (25.03%) | 0.000 (-0.013, 0.013) 0.992 |
|  | Q4 (225.00-2715.50 mg/day) | 1101 (25.03%) | 0.001 (-0.012, 0.014) 0.848 |

Income level, sedentary activity, calcium intake, and caffeine intake were divided into four groups according to the quartiles of distribution.

BMD, bone mineral density; BMI, body mass index; CCI, Charlson Comorbidity Index; CI, confidence interval; HMVPA, high moderate-to-vigorous physical activity; LMVPA, low moderate-to-vigorous physical activity; MMVPA, medium moderate-to-vigorous physical activity; MVPA, moderate-to-vigorous physical activity; NMVPA, no moderate-to-vigorous physical activity; PIR, poverty income ratio.

**Supplementary Table S3** Number of participants in each subgroup

| **Population** | **Group** | **Group 1**  **SD=7-8 h/day**  **(N)** | **Group 2**  **SD=6 h/day**  **(N)** | **Group 3**  **SD<6 h/day**  **(N)** | **Group 4**  **SD>8 h/day**  **(N)** |
| --- | --- | --- | --- | --- | --- |
| Overall | Total | 2371 | 1106 | 644 | 278 |
|  | VD intake <15 µg/day | 1682 | 824 | 521 | 206 |
|  | VD intake ≥ 15 µg/day | 689 | 282 | 123 | 72 |
|  | Deficient/Insufficient serum 25(OH)D | 1525 | 739 | 483 | 185 |
|  | Sufficient serum 25(OH)D | 846 | 367 | 161 | 93 |
| Aged <60 years | Total | 1426 | 711 | 422 | 133 |
|  | VD intake <15 µg/day | 1096 | 558 | 347 | 107 |
|  | VD intake ≥ 15 µg/day | 330 | 153 | 75 | 26 |
|  | Deficient/Insufficient serum 25(OH)D | 978 | 497 | 316 | 100 |
|  | Sufficient serum 25(OH)D | 448 | 214 | 106 | 33 |
| Aged ≥ 60 years | Total | 945 | 395 | 222 | 145 |
|  | VD intake <15 µg/day | 586 | 266 | 174 | 99 |
|  | VD intake ≥ 15 µg/day | 359 | 129 | 48 | 46 |
|  | Deficient/Insufficient serum 25(OH)D | 547 | 242 | 167 | 85 |
|  | Sufficient serum 25(OH)D | 398 | 153 | 55 | 60 |
| Men | Total | 1097 | 547 | 302 | 120 |
|  | VD intake <15 µg/day | 836 | 424 | 248 | 95 |
|  | VD intake ≥ 15 µg/day | 261 | 123 | 54 | 25 |
|  | Deficient/Insufficient serum 25(OH)D | 773 | 395 | 231 | 81 |
|  | Sufficient serum 25(OH)D | 324 | 152 | 71 | 39 |
| Non-postmenopausal women | Total | 444 | 202 | 114 | 56 |
|  | VD intake <15 µg/day | 332 | 154 | 93 | 45 |
|  | VD intake ≥ 15 µg/day | 112 | 48 | 21 | 11 |
|  | Deficient/Insufficient serum 25(OH)D | 286 | 137 | 87 | 41 |
|  | Sufficient serum 25(OH)D | 158 | 65 | 27 | 15 |
| Postmenopausal women | Total | 830 | 357 | 228 | 102 |
|  | VD intake <15 µg/day | 514 | 246 | 180 | 66 |
|  | VD intake ≥ 15 µg/day | 316 | 111 | 48 | 36 |
|  | Deficient/Insufficient serum 25(OH)D | 466 | 207 | 165 | 63 |
|  | Sufficient serum 25(OH)D | 364 | 150 | 63 | 39 |

SD, sleep duration; VD, vitamin D; 25(OH)D, 25-hydroxyvitamin D.

**Supplementary Table S4** Association between sleep duration and BMD

| **Population** | **Index** | **Group** | **Model 1** | **Model 2** | **Model 3** |
| --- | --- | --- | --- | --- | --- |
|  |  |  | **β (95% CI) P-value** | **β (95% CI) P-value** | **β (95% CI) P-value** |
| Total | TF-BMD | Group 1 | Reference (0) | Reference (0) | Reference (0) |
|  |  | Group 2 | **0.0121 (0.0010, 0.0232) 0.033233** | -0.0038 (-0.0126, 0.0050) 0.393510 | -0.0017 (-0.0104, 0.0070) 0.699505 |
|  |  | Group 3 | **0.0152 (0.0016, 0.0287) 0.028154** | -0.0095 (-0.0204, 0.0013) 0.084496 | -0.0026 (-0.0134, 0.0082) 0.636252 |
|  |  | Group 4 | **-0.0363 (-0.0556, -0.0169) 0.000239** | **-0.0286 (-0.0439, -0.0134) 0.000239** | **-0.0201 (-0.0352, -0.0050) 0.009000** |
|  | FN-BMD | Group 1 | Reference (0) | Reference (0) | Reference (0) |
|  |  | Group 2 | **0.0151 (0.0051, 0.0251) 0.003206** | 0.0002 (-0.0081, 0.0085) 0.962924 | 0.0021 (-0.0062, 0.0104) 0.620074 |
|  |  | Group 3 | **0.0189 (0.0066, 0.0311) 0.002540** | -0.0074 (-0.0177, 0.0028) 0.156793 | -0.0021 (-0.0124, 0.0081) 0.683777 |
|  |  | Group 4 | **-0.0297 (-0.0472, -0.0123) 0.000858** | **-0.0224 (-0.0369, -0.0080) 0.002337** | **-0.0158 (-0.0302, -0.0014) 0.031257** |
|  | TS-BMD | Group 1 | Reference (0) | Reference (0) | Reference (0) |
|  |  | Group 2 | 0.0058 (-0.0056, 0.0172) 0.316383 | -0.0041 (-0.0143, 0.0060) 0.422860 | -0.0037 (-0.0138, 0.0064) 0.469288 |
|  |  | Group 3 | 0.0046 (-0.0093, 0.0185) 0.515243 | -0.0123 (-0.0248, 0.0002) 0.052906 | -0.0094 (-0.0219, 0.0031) 0.142242 |
|  |  | Group 4 | -0.0155 (-0.0354, 0.0043) 0.125375 | -0.0172 (-0.0348, 0.0004) 0.054921 | -0.0137 (-0.0313, 0.0039) 0.126166 |
| Aged <60 years | TF-BMD | Group 1 | Reference (0) | Reference (0) | Reference (0) |
|  |  | Group 2 | **0.0160 (0.0028, 0.0291) 0.017531** | -0.0008 (-0.0116, 0.0100) 0.888738 | 0.0018 (-0.0089, 0.0125) 0.745407 |
|  |  | Group 3 | 0.0130 (-0.0029, 0.0289) 0.109672 | -0.0079 (-0.0211, 0.0052) 0.237347 | -0.0003 (-0.0134, 0.0129) 0.968598 |
|  |  | Group 4 | -0.0130 (-0.0390, 0.0130) 0.326926 | -0.0136 (-0.0349, 0.0077) 0.210919 | -0.0053 (-0.0264, 0.0158) 0.622540 |
|  | FN-BMD | Group 1 | Reference (0) | Reference (0) | Reference (0) |
|  |  | Group 2 | **0.0148 (0.0027, 0.0269) 0.016610** | 0.0011 (-0.0093, 0.0116) 0.834949 | 0.0033 (-0.0071, 0.0137) 0.534131 |
|  |  | Group 3 | **0.0160 (0.0014, 0.0306) 0.031492** | -0.0049 (-0.0177, 0.0078) 0.445727 | 0.0007 (-0.0120, 0.0135) 0.912616 |
|  |  | Group 4 | -0.0049 (-0.0287, 0.0190) 0.688639 | -0.0106 (-0.0311, 0.0100) 0.313040 | -0.0047 (-0.0251, 0.0158) 0.655399 |
|  | TS-BMD | Group 1 | Reference (0) | Reference (0) | Reference (0) |
|  |  | Group 2 | 0.0101 (-0.0030, 0.0232) 0.129914 | 0.0001 (-0.0117, 0.0120) 0.981224 | 0.0014 (-0.0105, 0.0133) 0.815263 |
|  |  | Group 3 | 0.0065 (-0.0093, 0.0223) 0.421309 | -0.0104 (-0.0249, 0.0040) 0.157543 | -0.0060 (-0.0206, 0.0086) 0.419069 |
|  |  | Group 4 | -0.0016 (-0.0274, 0.0243) 0.905930 | -0.0107 (-0.0341, 0.0127) 0.369678 | -0.0063 (-0.0297, 0.0171) 0.596512 |
| Aged ≥ 60 years | TF-BMD | Group 1 | Reference (0) | Reference (0) | Reference (0) |
|  |  | Group 2 | -0.0030 (-0.0216, 0.0156) 0.749736 | -0.0080 (-0.0230, 0.0069) 0.291931 | -0.0056 (-0.0203, 0.0092) 0.459618 |
|  |  | Group 3 | 0.0085 (-0.0146, 0.0316) 0.471113 | -0.0148 (-0.0338, 0.0042) 0.126997 | -0.0093 (-0.0281, 0.0095) 0.332938 |
|  |  | Group 4 | **-0.0412 (-0.0688, -0.0135) 0.003566** | **-0.0435 (-0.0657, -0.0214) 0.000118** | **-0.0347 (-0.0567, -0.0127) 0.002026** |
|  | FN-BMD | Group 1 | Reference (0) | Reference (0) | Reference (0) |
|  |  | Group 2 | 0.0064 (-0.0095, 0.0222) 0.430775 | -0.0014 (-0.0152, 0.0123) 0.840084 | 0.0010 (-0.0128, 0.0147) 0.891389 |
|  |  | Group 3 | 0.0118 (-0.0079, 0.0314) 0.242295 | -0.0144 (-0.0319, 0.0031) 0.106679 | -0.0092 (-0.0267, 0.0083) 0.302183 |
|  |  | Group 4 | **-0.0336 (-0.0571, -0.0100) 0.005262** | **-0.0342 (-0.0545, -0.0138) 0.001026** | **-0.0268 (-0.0473, -0.0064) 0.010113** |
|  | TS-BMD | Group 1 | Reference (0) | Reference (0) | Reference (0) |
|  |  | Group 2 | -0.0064 (-0.0271, 0.0144) 0.546788 | -0.0077 (-0.0260, 0.0105) 0.405318 | -0.0081 (-0.0263, 0.0101) 0.385189 |
|  |  | Group 3 | -0.0050 (-0.0308, 0.0208) 0.705684 | -0.0160 (-0.0391, 0.0072) 0.177028 | -0.0138 (-0.0371, 0.0094) 0.243469 |
|  |  | Group 4 | -0.0192 (-0.0501, 0.0117) 0.223493 | -0.0260 (-0.0530, 0.0010) 0.059105 | -0.0247 (-0.0518, 0.0025) 0.075016 |
| Men | TF-BMD | Group 1 | Reference (0) | Reference (0) | Reference (0) |
|  |  | Group 2 | 0.0067 (-0.0078, 0.0213) 0.366394 | -0.0048 (-0.0178, 0.0082) 0.468033 | -0.0029 (-0.0157, 0.0100) 0.663455 |
|  |  | Group 3 | 0.0088 (-0.0092, 0.0269) 0.337485 | **-0.0177 (-0.0341, -0.0012) 0.035406** | -0.0087 (-0.0252, 0.0078) 0.302542 |
|  |  | Group 4 | **-0.0367 (-0.0635, -0.0100) 0.007145** | **-0.0300 (-0.0539, -0.0061) 0.014022** | -0.0229 (-0.0466, 0.0008) 0.058054 |
|  | FN-BMD | Group 1 | Reference (0) | Reference (0) | Reference (0) |
|  |  | Group 2 | 0.0139 (-0.0001, 0.0278) 0.051455 | 0.0006 (-0.0118, 0.0130) 0.921438 | 0.0028 (-0.0095, 0.0151) 0.655316 |
|  |  | Group 3 | 0.0167 (-0.0006, 0.0340) 0.059011 | -0.0121 (-0.0278, 0.0036) 0.130363 | -0.0044 (-0.0202, 0.0114) 0.582598 |
|  |  | Group 4 | **-0.0340 (-0.0596, -0.0084) 0.009376** | **-0.0244 (-0.0471, -0.0016) 0.035790** | -0.0190 (-0.0417, 0.0037) 0.100698 |
|  | TS-BMD | Group 1 | Reference (0) | Reference (0) | Reference (0) |
|  |  | Group 2 | 0.0047 (-0.0111, 0.0204) 0.561388 | -0.0011 (-0.0161, 0.0138) 0.882342 | -0.0008 (-0.0157, 0.0141) 0.913179 |
|  |  | Group 3 | 0.0068 (-0.0127, 0.0264) 0.493255 | -0.0123 (-0.0312, 0.0066) 0.200850 | -0.0058 (-0.0249, 0.0132) 0.548271 |
|  |  | Group 4 | -0.0265 (-0.0554, 0.0024) 0.072586 | **-0.0337 (-0.0611, -0.0063) 0.016133** | **-0.0292 (-0.0566, -0.0019) 0.036506** |
| Non-postmenopausal  women | TF-BMD | Group 1 | Reference (0) | Reference (0) | Reference (0) |
|  |  | Group 2 | 0.0152 (-0.0067, 0.0370) 0.173319 | 0.0068 (-0.0122, 0.0258) 0.485705 | 0.0105 (-0.0084, 0.0295) 0.276851 |
|  |  | Group 3 | **0.0352 (0.0081, 0.0622) 0.010945** | 0.0089 (-0.0149, 0.0326) 0.463707 | 0.0141 (-0.0099, 0.0381) 0.248505 |
|  |  | Group 4 | -0.0007 (-0.0372, 0.0358) 0.968997 | -0.0060 (-0.0376, 0.0256) 0.708325 | 0.0027 (-0.0290, 0.0344) 0.867024 |
|  | FN-BMD | Group 1 | Reference (0) | Reference (0) | Reference (0) |
|  |  | Group 2 | 0.0139 (-0.0073, 0.0351) 0.199638 | 0.0049 (-0.0139, 0.0236) 0.611837 | 0.0081 (-0.0107, 0.0269) 0.399778 |
|  |  | Group 3 | **0.0357 (0.0095, 0.0620) 0.007743** | 0.0086 (-0.0148, 0.0320) 0.471061 | 0.0086 (-0.0152, 0.0324) 0.477993 |
|  |  | Group 4 | 0.0071 (-0.0283, 0.0425) 0.694563 | 0.0005 (-0.0307, 0.0317) 0.975115 | 0.0070 (-0.0245, 0.0384) 0.664627 |
|  | TS-BMD | Group 1 | Reference (0) | Reference (0) | Reference (0) |
|  |  | Group 2 | 0.0092 (-0.0128, 0.0311) 0.414420 | 0.0059 (-0.0149, 0.0266) 0.579512 | 0.0047 (-0.0162, 0.0255) 0.662099 |
|  |  | Group 3 | 0.0238 (-0.0034, 0.0510) 0.087294 | 0.0048 (-0.0212, 0.0307) 0.717669 | 0.0067 (-0.0197, 0.0331) 0.618004 |
|  |  | Group 4 | 0.0238 (-0.0129, 0.0605) 0.204610 | 0.0169 (-0.0177, 0.0514) 0.339468 | 0.0205 (-0.0144, 0.0554) 0.250586 |
| Postmenopausal  women | TF-BMD | Group 1 | Reference (0) | Reference (0) | Reference (0) |
|  |  | Group 2 | 0.0045 (-0.0128, 0.0219) 0.608905 | -0.0079 (-0.0231, 0.0072) 0.304164 | -0.0033 (-0.0183, 0.0117) 0.664068 |
|  |  | Group 3 | 0.0136 (-0.0069, 0.0340) 0.194587 | -0.0088 (-0.0268, 0.0093) 0.340999 | -0.0023 (-0.0203, 0.0156) 0.797777 |
|  |  | Group 4 | **-0.0457 (-0.0745, -0.0170) 0.001840** | **-0.0414 (-0.0664, -0.0163) 0.001260** | **-0.0306 (-0.0555, -0.0056) 0.016511** |
|  | FN-BMD | Group 1 | Reference (0) | Reference (0) | Reference (0) |
|  |  | Group 2 | 0.0092 (-0.0068, 0.0253) 0.260039 | -0.0037 (-0.0176, 0.0103) 0.607589 | 0.0004 (-0.0135, 0.0144) 0.954045 |
|  |  | Group 3 | 0.0143 (-0.0046, 0.0333) 0.139141 | -0.0094 (-0.0260, 0.0072) 0.267807 | -0.0042 (-0.0208, 0.0125) 0.624001 |
|  |  | Group 4 | **-0.0402 (-0.0669, -0.0136) 0.003093** | **-0.0328 (-0.0559, -0.0097) 0.005545** | **-0.0236 (-0.0468, -0.0004) 0.046158** |
|  | TS-BMD | Group 1 | Reference (0) | Reference (0) | Reference (0) |
|  |  | Group 2 | -0.0036 (-0.0229, 0.0157) 0.717099 | -0.0110 (-0.0288, 0.0067) 0.222958 | -0.0097 (-0.0275, 0.0081) 0.287272 |
|  |  | Group 3 | -0.0069 (-0.0297, 0.0160) 0.555576 | -0.0193 (-0.0404, 0.0018) 0.073186 | -0.0181 (-0.0394, 0.0031) 0.094982 |
|  |  | Group 4 | -0.0189 (-0.0509, 0.0131) 0.247042 | -0.0220 (-0.0514, 0.0074) 0.142235 | -0.0212 (-0.0508, 0.0085) 0.161676 |

Bold variables indicate P value < 0.05.

Model 1: unadjusted model; Model 2: age, sex/menopause status, race, and BMI were adjusted; Model 3: age, sex/menopause status, race, income level, BMI, smoking status, alcohol drinking status, physical activity level, fractures history, glucocorticoid use, family history of osteoporosis, CCI, calcium intake, vitamin D intake, and serum 25-hydroxyvitamin D were adjusted.

Group 1: SD=7-8 h/day; Group 2: SD=6 h/day; Group3: SD<6 h/day; Group 4: SD>8 h/day.

BMD, bone mineral density; BMI, body mass index; CCI, Charlson Comorbidity Index; CI, confidence interval; FN, femoral neck; SD, sleep duration; TF, total femur; TS, total spine.

**Supplementary Table S5** Association between sleep duration and BMD in different vitamin D intake levels

| **Population** | **Model** | **Group** | **TF-BMD** | | **FN-BMD** | | **TS-BMD** | |
| --- | --- | --- | --- | --- | --- | --- | --- | --- |
|  |  |  | **Inadequate**  **VD intake** | **Adequate**  **VD intake** | **Inadequate**  **VD intake** | **Adequate**  **VD intake** | **Inadequate**  **VD intake** | **Adequate**  **VD intake** |
|  |  |  | **β (95% CI) P-value** | **β (95% CI) P-value** | **β (95% CI) P-value** | **β (95% CI) P-value** | **β (95% CI) P-value** | **β (95% CI) P-value** |
| Total | Model 1 | Group 1 | Reference (0) | Reference (0) | Reference (0) | Reference (0) | Reference (0) | Reference (0) |
|  |  | Group 2 | 0.0087  (-0.0043, 0.0216)  0.188925 | 0.0167  (-0.0048, 0.0382)  0.128036 | 0.0111  (-0.0006, 0.0227)  0.062279 | **0.0216**  **(0.0021, 0.0411)**  **0.030381** | 0.0034  (-0.0098, 0.0165)  0.612657 | 0.0090  (-0.0137, 0.0317)  0.437036 |
|  |  | Group 3 | 0.0050  (-0.0102, 0.0202)  0.517948 | **0.0383**  **(0.0085, 0.0681)**  **0.011808** | 0.0091  (-0.0046, 0.0229)  0.191917 | **0.0404**  **(0.0134, 0.0674)**  **0.003459** | -0.0023  (-0.0178, 0.0132)  0.767149 | 0.0195  (-0.0120, 0.0509)  0.225357 |
|  |  | Group 4 | **-0.0382**  **(-0.0606, -0.0158)**  **0.000837** | -0.0353  (-0.0730, 0.0024)  0.066442 | **-0.0331**  **(-0.0533, -0.0129)**  **0.001352** | -0.0249  (-0.0590, 0.0093)  0.154609 | -0.0174  (-0.0402, 0.0054)  0.135238 | -0.0136  (-0.0534, 0.0262)  0.504013 |
|  | Model 2 | Group 1 | Reference (0) | Reference (0) | Reference (0) | Reference (0) | Reference (0) | Reference (0) |
|  |  | Group 2 | -0.0032  (-0.0135, 0.0072)  0.548157 | -0.0042  (-0.0208, 0.0125)  0.625690 | -0.0001  (-0.0099, 0.0096)  0.976636 | 0.0027  (-0.0133, 0.0186)  0.743583 | -0.0038  (-0.0155, 0.0080)  0.529544 | -0.0040  (-0.0241, 0.0160)  0.692522 |
|  |  | Group 3 | -0.0105  (-0.0228, 0.0018)  0.093914 | -0.0022  (-0.0256, 0.0211)  0.852242 | -0.0095  (-0.0211, 0.0022)  0.110987 | 0.0018  (-0.0205, 0.0241)  0.876027 | -0.0137  (-0.0277, 0.0002)  0.053999 | -0.0078  (-0.0359, 0.0202)  0.584091 |
|  |  | Group 4 | **-0.0273**  **(-0.0452, -0.0094)**  **0.002857** | **-0.0303**  **(-0.0594, -0.0013)**  **0.040732** | **-0.0229**  **(-0.0398, -0.0059)**  **0.008176** | -0.0206  (-0.0484, 0.0071)  0.144779 | -0.0178  (-0.0381, 0.0026)  0.086601 | -0.0156  (-0.0505, 0.0193)  0.381481 |
|  | Model 3 | Group 1 | Reference (0) | Reference (0) | Reference (0) | Reference (0) | Reference (0) | Reference (0) |
|  |  | Group 2 | -0.0012  (-0.0114, 0.0091)  0.823860 | -0.0002  (-0.0167, 0.0163)  0.979432 | 0.0017  (-0.0080, 0.0114)  0.736061 | 0.0051  (-0.0109, 0.0210)  0.534312 | -0.0027  (-0.0144, 0.0090)  0.655071 | -0.0041  (-0.0242, 0.0159)  0.686347 |
|  |  | Group 3 | -0.0036  (-0.0158, 0.0087)  0.569782 | 0.0038  (-0.0194, 0.0270)  0.746484 | -0.0038  (-0.0154, 0.0079)  0.525498 | 0.0049  (-0.0175, 0.0273)  0.668510 | -0.0103  (-0.0243, 0.0037)  0.150613 | -0.0053  (-0.0335, 0.0229)  0.711078 |
|  |  | Group 4 | **-0.0198**  **(-0.0376, -0.0020)**  **0.029505** | -0.0204  (-0.0492, 0.0083)  0.163579 | -0.0164  (-0.0333, 0.0004)  0.056490 | -0.0132  (-0.0409, 0.0146)  0.352982 | -0.0140  (-0.0343, 0.0064)  0.179574 | -0.0134  (-0.0483, 0.0216)  0.452945 |
| Aged <60 years | Model 1 | Group 1 | Reference (0) | Reference (0) | Reference (0) | Reference (0) | Reference (0) | Reference (0) |
|  |  | Group 2 | 0.0140  (-0.0009, 0.0289)  0.064897 | 0.0214  (-0.0067, 0.0495)  0.136293 | 0.0125  (-0.0011, 0.0262)  0.071178 | 0.0212  (-0.0048, 0.0473)  0.111156 | 0.0079  (-0.0068, 0.0226)  0.291070 | 0.0170  (-0.0115, 0.0456)  0.242135 |
|  |  | Group 3 | 0.0053  (-0.0123, 0.0229)  0.556286 | **0.0420**  **(0.0053, 0.0788)**  **0.025431** | 0.0085  (-0.0076, 0.0246)  0.301271 | **0.0440**  **(0.0099, 0.0780)**  **0.011722** | -0.0005  (-0.0179, 0.0169)  0.954354 | 0.0348  (-0.0026, 0.0721)  0.068540 |
|  |  | Group 4 | -0.0168  (-0.0458, 0.0122)  0.256527 | -0.0014  (-0.0599, 0.0572)  0.963835 | -0.0109  (-0.0374, 0.0156)  0.421623 | 0.0157  (-0.0386, 0.0700)  0.570493 | -0.0021  (-0.0308, 0.0266)  0.885159 | -0.0017  (-0.0612, 0.0577)  0.954191 |
|  | Model 2 | Group 1 | Reference (0) | Reference (0) | Reference (0) | Reference (0) | Reference (0) | Reference (0) |
|  |  | Group 2 | 0.0005  (-0.0118, 0.0129)  0.933421 | -0.0048  (-0.0273, 0.0177)  0.675618 | 0.0018  (-0.0101, 0.0137)  0.770317 | -0.0013  (-0.0233, 0.0207)  0.908191 | 0.0009  (-0.0126, 0.0144)  0.895346 | -0.0025  (-0.0279, 0.0229)  0.846497 |
|  |  | Group 3 | -0.0106  (-0.0254, 0.0041)  0.158012 | 0.0049  (-0.0248, 0.0345)  0.748505 | -0.0088  (-0.0230, 0.0054)  0.225861 | 0.0104  (-0.0186, 0.0394)  0.481259 | -0.0147 (-0.0307, 0.0014) 0.073613 | 0.0064  (-0.0271, 0.0399)  0.707149 |
|  |  | Group 4 | -0.0135  (-0.0375, 0.0104)  0.268879 | -0.0113  (-0.0579, 0.0354)  0.635141 | -0.0135  (-0.0366, 0.0096)  0.252031 | 0.0022  (-0.0434, 0.0477)  0.926003 | -0.0074 (-0.0335, 0.0187) 0.579372 | -0.0237  (-0.0763, 0.0289)  0.378408 |
|  | Model 3 | Group 1 | Reference (0) | Reference (0) | Reference (0) | Reference (0) | Reference (0) | Reference (0) |
|  |  | Group 2 | 0.0027  (-0.0096, 0.0149)  0.668809 | -0.0015  (-0.0241, 0.0212)  0.899150 | 0.0033  (-0.0085, 0.0152)  0.579496 | 0.0002  (-0.0220, 0.0224)  0.984017 | 0.0025  (-0.0109, 0.0160)  0.713678 | -0.0021  (-0.0277, 0.0236)  0.875259 |
|  |  | Group 3 | -0.0028  (-0.0174, 0.0119)  0.713477 | 0.0122  (-0.0178, 0.0422)  0.426007 | -0.0027  (-0.0170, 0.0115)  0.705895 | 0.0161  (-0.0133, 0.0456)  0.283311 | -0.0100  (-0.0262, 0.0062)  0.224901 | 0.0108  (-0.0231, 0.0448)  0.532176 |
|  |  | Group 4 | -0.0065  (-0.0303, 0.0172)  0.589391 | 0.0004  (-0.0469, 0.0477)  0.987849 | -0.0086  (-0.0316, 0.0144)  0.462275 | 0.0122  (-0.0342, 0.0586)  0.606448 | -0.0045  (-0.0307, 0.0216)  0.734348 | -0.0181  (-0.0717, 0.0354)  0.506938 |
| Aged ≥ 60 years | Model 1 | Group 1 | Reference (0) | Reference (0) | Reference (0) | Reference (0) | Reference (0) | Reference (0) |
|  |  | Group 2 | -0.0076  (-0.0308, 0.0157)  0.523417 | 0.0010  (-0.0297, 0.0317)  0.950627 | 0.0020  (-0.0177, 0.0217)  0.841011 | 0.0109  (-0.0156, 0.0374)  0.419779 | -0.0085  (-0.0343, 0.0173)  0.519063 | -0.0072  (-0.0420, 0.0276)  0.685189 |
|  |  | Group 3 | 0.0018  (-0.0253, 0.0289)  0.897441 | 0.0080  (-0.0380, 0.0539)  0.734306 | 0.0071  (-0.0158, 0.0301)  0.543038 | 0.0080  (-0.0316, 0.0476)  0.693006 | -0.0073  (-0.0374, 0.0228)  0.635113 | -0.0205  (-0.0726, 0.0316)  0.440479 |
|  |  | Group 4 | **-0.0443**  **(-0.0784, -0.0101)**  **0.011158** | -0.0410  (-0.0878, 0.0059)  0.086887 | **-0.0363**  **(-0.0652, -0.0074)**  **0.013998** | -0.0330  (-0.0734, 0.0074)  0.109493 | -0.0257  (-0.0636, 0.0122)  0.183939 | -0.0114  (-0.0645, 0.0417)  0.673785 |
|  | Model 2 | Group 1 | Reference (0) | Reference (0) | Reference (0) | Reference (0) | Reference (0) | Reference (0) |
|  |  | Group 2 | -0.0095  (-0.0282, 0.0092)  0.321588 | -0.0043  (-0.0291, 0.0206)  0.737686 | -0.0038  (-0.0209, 0.0134)  0.666413 | 0.0047  (-0.0185, 0.0280)  0.689864 | -0.0102  (-0.0325, 0.0121)  0.371027 | -0.0031  (-0.0347, 0.0284)  0.847292 |
|  |  | Group 3 | -0.0122  (-0.0345, 0.0101)  0.282439 | -0.0176  (-0.0552, 0.0200)  0.360280 | -0.0130  (-0.0334, 0.0074)  0.212304 | -0.0168  (-0.0520, 0.0183)  0.348391 | -0.0120  (-0.0386, 0.0145)  0.374296 | -0.0258  (-0.0735, 0.0219)  0.289091 |
|  |  | Group 4 | **-0.0441**  **(-0.0716, -0.0167)**  **0.001666** | **-0.0439**  **(-0.0814, -0.0065)**  **0.021888** | **-0.0335**  **(-0.0586, -0.0083)**  **0.009147** | **-0.0362**  **(-0.0712, -0.0011)**  **0.043485** | **-0.0329**  **(-0.0656, -0.0002)**  **0.048869** | -0.0143  (-0.0618, 0.0332)  0.556025 |
|  | Model 3 | Group 1 | Reference (0) | Reference (0) | Reference (0) | Reference (0) | Reference (0) | Reference (0) |
|  |  | Group 2 | -0.0075  (-0.0261, 0.0110)  0.426640 | -0.0016  (-0.0264, 0.0231)  0.897355 | -0.0010  (-0.0181, 0.0162)  0.909659 | 0.0054  (-0.0181, 0.0289)  0.653376 | -0.0100  (-0.0324, 0.0124)  0.381559 | -0.0027  (-0.0348, 0.0294)  0.867923 |
|  |  | Group 3 | -0.0086  (-0.0308, 0.0136)  0.446968 | -0.0071  (-0.0446, 0.0305)  0.712454 | -0.0087  (-0.0292, 0.0118)  0.405639 | -0.0103  (-0.0459, 0.0253)  0.570810 | -0.0098  (-0.0366, 0.0169)  0.471270 | -0.0208  (-0.0694, 0.0279)  0.403385 |
|  |  | Group 4 | **-0.0370**  **(-0.0645, -0.0094)**  **0.008743** | -0.0341  (-0.0710, 0.0028)  0.070509 | **-0.0259**  **(-0.0514, -0.0004)**  **0.046707** | -0.0287  (-0.0637, 0.0063)  0.108775 | -0.0316  (-0.0649, 0.0017)  0.063264 | -0.0123  (-0.0600, 0.0355)  0.615506 |
| Men | Model 1 | Group 1 | Reference (0) | Reference (0) | Reference (0) | Reference (0) | Reference (0) | Reference (0) |
|  |  | Group 2 | 0.0089  (-0.0077, 0.0255)  0.293784 | -0.0017  (-0.0321, 0.0287)  0.914209 | 0.0141  (-0.0017, 0.0299)  0.081113 | 0.0119  (-0.0175, 0.0413)  0.426855 | 0.0039  (-0.0138, 0.0216)  0.666933 | 0.0067  (-0.0278, 0.0413)  0.703236 |
|  |  | Group 3 | 0.0046  (-0.0155, 0.0247)  0.651522 | 0.0231  (-0.0184, 0.0646)  0.275917 | 0.0098  (-0.0094, 0.0290)  0.315964 | 0.0416  (0.0015, 0.0818)  0.042807 | 0.0024  (-0.0191, 0.0238)  0.829383 | 0.0237  (-0.0235, 0.0709)  0.326046 |
|  |  | Group 4 | **-0.0420**  **(-0.0721, -0.0119)**  **0.006362** | -0.0190  (-0.0772, 0.0391)  0.521231 | **-0.0429**  **(-0.0716, -0.0142)**  **0.003444** | -0.0027  (-0.0590, 0.0535)  0.924170 | **-0.0368**  **(-0.0689, -0.0047)**  **0.024750** | 0.0110  (-0.0551, 0.0772)  0.743667 |
|  | Model 2 | Group 1 | Reference (0) | Reference (0) | Reference (0) | Reference (0) | Reference (0) | Reference (0) |
|  |  | Group 2 | -0.0002  (-0.0152, 0.0147)  0.974603 | -0.0199  (-0.0465, 0.0067)  0.142532 | 0.0039  (-0.0103, 0.0181)  0.591180 | -0.0103  (-0.0357, 0.0151)  0.426807 | -0.0004  (-0.0171, 0.0162)  0.958153 | -0.0023  (-0.0360, 0.0313)  0.891029 |
|  |  | Group 3 | -0.0160  (-0.0344, 0.0025)  0.090541 | -0.0262  (-0.0634, 0.0109)  0.167287 | -0.0128  (-0.0303, 0.0048)  0.154658 | -0.0118  (-0.0473, 0.0236)  0.513888 | -0.0147  (-0.0352, 0.0059)  0.162265 | -0.0075  (-0.0545, 0.0395)  0.754076 |
|  |  | Group 4 | **-0.0324**  **(-0.0597, -0.0052)**  **0.019661** | -0.0203  (-0.0708, 0.0302)  0.431158 | **-0.0303**  **(-0.0562, -0.0044)**  **0.022123** | -0.0036  (-0.0518, 0.0446)  0.885115 | **-0.0426**  **(-0.0730, -0.0123)**  **0.005906** | 0.0006  (-0.0632, 0.0645)  0.984743 |
|  | Model 3 | Group 1 | Reference (0) | Reference (0) | Reference (0) | Reference (0) | Reference (0) | Reference (0) |
|  |  | Group 2 | 0.0015  (-0.0133, 0.0164)  0.838671 | -0.0147  (-0.0413, 0.0119)  0.279409 | 0.0056  (-0.0085, 0.0198)  0.435782 | -0.0068  (-0.0324, 0.0189)  0.604695 | -0.0007  (-0.0173, 0.0159)  0.932897 | 0.0020  (-0.0321, 0.0362)  0.906628 |
|  |  | Group 3 | -0.0065  (-0.0251, 0.0120)  0.488781 | -0.0180  (-0.0554, 0.0194)  0.345805 | -0.0047  (-0.0224, 0.0130)  0.604890 | -0.0072  (-0.0432, 0.0289)  0.697390 | -0.0078  (-0.0285, 0.0130)  0.462025 | -0.0052  (-0.0532, 0.0427)  0.831141 |
|  |  | Group 4 | -0.0268  (-0.0538, 0.0002)  0.052112 | -0.0120  (-0.0632, 0.0393)  0.647549 | **-0.0262**  **(-0.0520, -0.0004)**  **0.046381** | 0.0002  (-0.0492, 0.0495)  0.994198 | **-0.0409**  **(-0.0711, -0.0106)**  **0.008170** | 0.0008  (-0.0648, 0.0665)  0.979872 |
| Non-postmenopausal women | Model 1 | Group 1 | Reference (0) | Reference (0) | Reference (0) | Reference (0) | Reference (0) | Reference (0) |
|  |  | Group 2 | 0.0176  (-0.0083, 0.0434)  0.183776 | 0.0079  (-0.0321, 0.0479)  0.699200 | 0.0149  (-0.0099, 0.0398)  0.239619 | 0.0098  (-0.0303, 0.0499)  0.632281 | 0.0149  (-0.0108, 0.0405)  0.256099 | -0.0089  (-0.0514, 0.0336)  0.681302 |
|  |  | Group 3 | **0.0317**  **(0.0006, 0.0628)**  **0.046353** | 0.0524  (-0.0028, 0.1075)  0.064211 | 0.0282  (-0.0017, 0.0582)  0.065350 | **0.0650**  **(0.0097, 0.1203)**  **0.022358** | 0.0218  (-0.0091, 0.0527)  0.166881 | 0.0340  (-0.0246, 0.0926)  0.257113 |
|  |  | Group 4 | 0.0063  (-0.0358, 0.0484)  0.769807 | -0.0280  (-0.1013, 0.0452)  0.453894 | 0.0052  (-0.0354, 0.0457)  0.802288 | 0.0118  (-0.0617, 0.0853)  0.752857 | 0.0349  (-0.0069, 0.0767)  0.101825 | -0.0206  (-0.0985, 0.0573)  0.604711 |
|  | Model 2 | Group 1 | Reference (0) | Reference (0) | Reference (0) | Reference (0) | Reference (0) | Reference (0) |
|  |  | Group 2 | 0.0095  (-0.0129, 0.0319)  0.405088 | 0.0003  (-0.0350, 0.0355)  0.988829 | 0.0068  (-0.0151, 0.0287)  0.544241 | 0.0009  (-0.0355, 0.0374)  0.960138 | 0.0120  (-0.0121, 0.0362)  0.329294 | -0.0103  (-0.0507, 0.0301)  0.617811 |
|  |  | Group 3 | 0.0084  (-0.0187, 0.0356)  0.543258 | 0.0170  (-0.0332, 0.0672)  0.507259 | 0.0046  (-0.0219, 0.0312)  0.733141 | 0.0283  (-0.0236, 0.0802)  0.286587 | 0.0045  (-0.0248, 0.0338)  0.764058 | 0.0093  (-0.0482, 0.0669)  0.750656 |
|  |  | Group 4 | 0.0024  (-0.0340, 0.0388)  0.897115 | -0.0384  (-0.1034, 0.0266)  0.248568 | 0.0006  (-0.0350, 0.0361)  0.973909 | 0.0028  (-0.0644, 0.0700)  0.935287 | 0.0289  (-0.0103, 0.0681)  0.148912 | -0.0298  (-0.1043, 0.0447)  0.433914 |
|  | Model 3 | Group 1 | Reference (0) | Reference (0) | Reference (0) | Reference (0) | Reference (0) | Reference (0) |
|  |  | Group 2 | 0.0140  (-0.0083, 0.0364)  0.219524 | -0.0021  (-0.0394, 0.0351)  0.910513 | 0.0093  (-0.0127, 0.0313)  0.409490 | 0.0045  (-0.0334, 0.0425)  0.815114 | 0.0112  (-0.0129, 0.0354)  0.362677 | -0.0204  (-0.0628, 0.0221)  0.348286 |
|  |  | Group 3 | 0.0145  (-0.0128, 0.0418)  0.299566 | 0.0191  (-0.0362, 0.0745)  0.499122 | 0.0065  (-0.0204, 0.0334)  0.636570 | 0.0261  (-0.0302, 0.0825)  0.365098 | 0.0068  (-0.0228, 0.0363)  0.653841 | 0.0028  (-0.0603, 0.0658)  0.930996 |
|  |  | Group 4 | 0.0110  (-0.0255, 0.0475)  0.555126 | -0.0238  (-0.0935, 0.0458)  0.503753 | 0.0034  (-0.0326, 0.0393)  0.854476 | 0.0328  (-0.0381, 0.1038)  0.366141 | 0.0313  (-0.0081, 0.0708)  0.119892 | -0.0273  (-0.1067, 0.0520)  0.500645 |
| Postmenopausal women | Model 1 | Group 1 | Reference (0) | Reference (0) | Reference (0) | Reference (0) | Reference (0) | Reference (0) |
|  |  | Group 2 | -0.0031  (-0.0243, 0.0181)  0.771735 | 0.0153  (-0.0149, 0.0454)  0.321370 | 0.0011  (-0.0186, 0.0207)  0.916666 | 0.0202  (-0.0075, 0.0478)  0.153439 | -0.0075  (-0.0311, 0.0162)  0.537236 | 0.0007  (-0.0329, 0.0343)  0.968754 |
|  |  | Group 3 | 0.0053  (-0.0184, 0.0290)  0.662562 | 0.0221  (-0.0202, 0.0644)  0.306125 | 0.0086  (-0.0134, 0.0305)  0.444497 | 0.0100  (-0.0288, 0.0488)  0.613982 | -0.0098  (-0.0363, 0.0166)  0.465732 | -0.0114  (-0.0586, 0.0358)  0.636180 |
|  |  | Group 4 | **-0.0520**  **(-0.0877, -0.0162)**  **0.004474** | -0.0365  (-0.0845, 0.0116)  0.137466 | **-0.0402**  **(-0.0734, -0.0071)**  **0.017592** | -0.0428  (-0.0869, 0.0013)  0.057851 | -0.0196  (-0.0596, 0.0203)  0.335545 | -0.0191  (-0.0727, 0.0344)  0.484037 |
|  | Model 2 | Group 1 | Reference (0) | Reference (0) | Reference (0) | Reference (0) | Reference (0) | Reference (0) |
|  |  | Group 2 | -0.0159  (-0.0343, 0.0025)  0.091372 | 0.0062  (-0.0209, 0.0332)  0.655423 | -0.0116  (-0.0285, 0.0054)  0.181028 | 0.0118  (-0.0132, 0.0369)  0.354178 | -0.0178  (-0.0395, 0.0039)  0.107437 | -0.0022  (-0.0334, 0.0290)  0.890430 |
|  |  | Group 3 | -0.0141  (-0.0348, 0.0065)  0.180458 | 0.0061  (-0.0316, 0.0439)  0.749804 | -0.0123  (-0.0313, 0.0067)  0.203184 | -0.0049  (-0.0399, 0.0301)  0.783041 | -0.0223  (-0.0466, 0.0020)  0.072340 | -0.0170  (-0.0606, 0.0266)  0.445652 |
|  |  | Group 4 | **-0.0448**  **(-0.0760, -0.0137)**  **0.004826** | -0.0372  (-0.0798, 0.0054)  0.087548 | **-0.0296**  **(-0.0582, -0.0010)**  **0.042845** | **-0.0417**  **(-0.0812, -0.0022)**  **0.038960** | -0.0248  (-0.0614, 0.0118)  0.184140 | -0.0223  (-0.0715, 0.0269)  0.375514 |
|  | Model 3 | Group 1 | Reference (0) | Reference (0) | Reference (0) | Reference (0) | Reference (0) | Reference (0) |
|  |  | Group 2 | -0.0106  (-0.0289, 0.0078)  0.259899 | 0.0068  (-0.0199, 0.0336)  0.617297 | -0.0058  (-0.0228, 0.0112)  0.502632 | 0.0103  (-0.0150, 0.0355)  0.424662 | -0.0154  (-0.0373, 0.0066)  0.169939 | -0.0043  (-0.0357, 0.0272)  0.788929 |
|  |  | Group 3 | -0.0078  (-0.0285, 0.0128)  0.457259 | 0.0175  (-0.0203, 0.0553)  0.364023 | -0.0063  (-0.0254, 0.0127)  0.515124 | 0.0020  (-0.0336, 0.0377)  0.911769 | -0.0210  (-0.0457, 0.0036)  0.094799 | -0.0104  (-0.0548, 0.0340)  0.646744 |
|  |  | Group 4 | **-0.0350**  **(-0.0662, -0.0037)**  **0.028548** | -0.0358  (-0.0782, 0.0067)  0.099344 | -0.0197  (-0.0485, 0.0092)  0.182274 | **-0.0408**  **(-0.0809, -0.0008)**  **0.046284** | -0.0234  (-0.0607, 0.0139)  0.219983 | -0.0234  (-0.0733, 0.0265)  0.358521 |

Bold variables indicate P value < 0.05.

Model 1: unadjusted model; Model 2: age, sex/menopause status, race, and BMI were adjusted; Model 3: age, sex/menopause status, race, income level, BMI, smoking status, alcohol drinking status, physical activity level, fractures history, glucocorticoid use, family history of osteoporosis, CCI, calcium intake, and serum 25-hydroxyvitamin D were adjusted.

Group 1: SD=7-8 h/day; Group 2: SD=6 h/day; Group3: SD<6 h/day; Group 4: SD>8 h/day.

BMD, bone mineral density; BMI, body mass index; CCI, Charlson Comorbidity Index; CI, confidence interval; FN, femoral neck; SD, sleep duration; TF, total femur; TS, total spine; VD, vitamin D.

**Supplementary Table S6** Association between sleep duration and BMD in different serum 25(OH) levels

| **Population** | **Model** | **Group** | **TF-BMD** | | **FN-BMD** | | **TS-BMD** | |
| --- | --- | --- | --- | --- | --- | --- | --- | --- |
|  |  |  | **Deficient/Insufficient**  **serum 25(OH)D** | **Sufficient**  **serum 25(OH)D** | **Deficient/Insufficient**  **serum 25(OH)D** | **Sufficient**  **serum 25(OH)D** | **Deficient/Insufficient**  **serum 25(OH)D** | **Sufficient**  **serum 25(OH)D** |
|  |  |  | **β (95% CI) P-value** | **β (95% CI) P-value** | **β (95% CI) P-value** | **β (95% CI) P-value** | **β (95% CI) P-value** | **β (95% CI) P-value** |
| Total | Model 1 | Group 1 | Reference (0) | Reference (0) | Reference (0) | Reference (0) | Reference (0) | Reference (0) |
|  |  | Group 2 | 0.0122  (-0.0013, 0.0258)  0.076912 | 0.0082  (-0.0106, 0.0271) 0.393117 | 0.0149  (0.0025, 0.0273)  0.018596 | 0.0124  (-0.0042, 0.0290)  0.144467 | 0.0115  (-0.0024, 0.0254)  0.105230 | -0.0071  (-0.0269, 0.0128)  0.486435 |
|  |  | Group 3 | 0.0035  (-0.0123, 0.0193)  0.663583 | **0.0304**  **(0.0045, 0.0564)**  **0.021692** | 0.0116  (-0.0028, 0.0260)  0.115503 | **0.0230**  **(0.0001, 0.0459)**  **0.049027** | -0.0001  (-0.0162, 0.0161)  0.994166 | 0.0100  (-0.0173, 0.0374)  0.471839 |
|  |  | Group 4 | **-0.0402**  **(-0.0638, -0.0166)**  **0.000836** | -0.0315  (-0.0645, 0.0014)  0.061134 | **-0.0314**  **(-0.0530, -0.0099)**  **0.004233** | -0.0291  (-0.0582, -0.0000)  0.050157 | -0.0184  (-0.0425, 0.0057)  0.135510 | -0.0112  (-0.0459, 0.0235)  0.526656 |
|  | Model 2 | Group 1 | Reference (0) | Reference (0) | Reference (0) | Reference (0) | Reference (0) | Reference (0) |
|  |  | Group 2 | -0.0008  (-0.0117, 0.0100)  0.883678 | -0.0096  (-0.0246, 0.0054)  0.211515 | 0.0020  (-0.0084, 0.0123)  0.711325 | -0.0030  (-0.0170, 0.0110)  0.675071 | 0.0034  (-0.0090, 0.0158)  0.591543 | **-0.0185**  **(-0.0362, -0.0008)**  **0.040912** |
|  |  | Group 3 | -0.0112  (-0.0240, 0.0016)  0.085426 | -0.0025  (-0.0233, 0.0184)  0.817555 | -0.0072  (-0.0194, 0.0050) 0.245512 | -0.0067  (-0.0261, 0.0128)  0.502699 | -0.0114  (-0.0260, 0.0031)  0.123797 | -0.0104  (-0.0349, 0.0141)  0.404911 |
|  |  | Group 4 | **-0.0289**  **(-0.0477, -0.0101)**  **0.002634** | **-0.0266**  **(-0.0528, -0.0005)**  **0.046148** | **-0.0224**  **(-0.0404, -0.0045)**  **0.014240** | -0.0215  (-0.0459, 0.0029)  0.084883 | -0.0188  (-0.0403, 0.0026)  0.085319 | -0.0139  (-0.0446, 0.0169)  0.377885 |
|  | Model 3 | Group 1 | Reference (0) | Reference (0) | Reference (0) | Reference (0) | Reference (0) | Reference (0) |
|  |  | Group 2 | 0.0007  (-0.0100, 0.0115)  0.894240 | -0.0054  (-0.0202, 0.0093)  0.468801 | 0.0035  (-0.0068, 0.0138)  0.502692 | 0.0005  (-0.0134, 0.0143)  0.947574 | 0.0039  (-0.0084, 0.0163)  0.534749 | **-0.0177**  **(-0.0355, -0.0000)**  **0.049760** |
|  |  | Group 3 | -0.0068  (-0.0196, 0.0059)  0.293965 | 0.0121  (-0.0084, 0.0327)  0.248280 | -0.0040  (-0.0162, 0.0082)  0.523820 | 0.0045  (-0.0149, 0.0239)  0.647578 | -0.0098  (-0.0244, 0.0048)  0.187317 | -0.0048  (-0.0295, 0.0200)  0.706114 |
|  |  | Group 4 | **-0.0223**  **(-0.0410, -0.0036)**  **0.019455** | -0.0140  (-0.0398, 0.0118)  0.287628 | -0.0173  (-0.0352, 0.0006)  0.058042 | -0.0113  (-0.0357, 0.0130)  0.361720 | -0.0155  (-0.0369, 0.0060)  0.157613 | -0.0102  (-0.0413, 0.0209)  0.519808 |
| Aged <60 years | Model 1 | Group 1 | Reference (0) | Reference (0) | Reference (0) | Reference (0) | Reference (0) | Reference (0) |
|  |  | Group 2 | **0.0168**  **(0.0012, 0.0325)**  **0.035264** | 0.0122  (-0.0116, 0.0360)  0.314826 | **0.0176**  **(0.0030, 0.0321)**  **0.018242** | 0.0068  (-0.0143, 0.0279)  0.526694 | **0.0185**  **(0.0028, 0.0341)**  **0.020990** | -0.0099  (-0.0335, 0.0136)  0.408910 |
|  |  | Group 3 | 0.0064  (-0.0120, 0.0248)  0.493232 | 0.0225  (-0.0084, 0.0535)  0.153364 | 0.0130  (-0.0041, 0.0301)  0.136241 | 0.0165  (-0.0110, 0.0439)  0.239725 | 0.0046  (-0.0138, 0.0230)  0.624445 | 0.0084  (-0.0222, 0.0391)  0.589596 |
|  |  | Group 4 | -0.0215  (-0.0513, 0.0084)  0.158746 | 0.0021  (-0.0495, 0.0538)  0.936158 | -0.0124  (-0.0401, 0.0154)  0.382893 | 0.0088  (-0.0370, 0.0546)  0.706671 | -0.0019  (-0.0317, 0.0280)  0.902443 | -0.0045  (-0.0557, 0.0467)  0.863476 |
|  | Model 2 | Group 1 | Reference (0) | Reference (0) | Reference (0) | Reference (0) | Reference (0) | Reference (0) |
|  |  | Group 2 | -0.0008  (-0.0139, 0.0122)  0.899955 | -0.0013  (-0.0208, 0.0182)  0.898774 | 0.0024  (-0.0103, 0.0151)  0.708428 | -0.0022  (-0.0206, 0.0161)  0.811146 | 0.0079  (-0.0065, 0.0224)  0.279725 | -0.0168  (-0.0378, 0.0043)  0.119519 |
|  |  | Group 3 | -0.0119  (-0.0273, 0.0036)  0.132025 | 0.0028  (-0.0226, 0.0283)  0.827980 | -0.0072  (-0.0222, 0.0078)  0.348573 | 0.0014  (-0.0225, 0.0253)  0.910627 | -0.0127  (-0.0297, 0.0043)  0.144488 | -0.0021  (-0.0296, 0.0253)  0.878360 |
|  |  | Group 4 | -0.0186  (-0.0433, 0.0062)  0.141296 | 0.0041  (-0.0382, 0.0463)  0.850169 | -0.0147  (-0.0388, 0.0094)  0.232023 | 0.0059  (-0.0338, 0.0456)  0.770442 | -0.0108  (-0.0382, 0.0165)  0.436632 | -0.0107  (-0.0564, 0.0349)  0.644382 |
|  | Model 3 | Group 1 | Reference (0) | Reference (0) | Reference (0) | Reference (0) | Reference (0) | Reference (0) |
|  |  | Group 2 | 0.0008  (-0.0122, 0.0137)  0.908226 | 0.0038  (-0.0156, 0.0232)  0.699545 | 0.0041  (-0.0086, 0.0168)  0.525006 | 0.0020  (-0.0163, 0.0204)  0.828102 | 0.0087  (-0.0057, 0.0231)  0.236070 | -0.0172  (-0.0385, 0.0042)  0.115204 |
|  |  | Group 3 | -0.0059  (-0.0213, 0.0095)  0.455575 | 0.0168  (-0.0087, 0.0424)  0.196542 | -0.0031  (-0.0182, 0.0120)  0.684573 | 0.0118  (-0.0124, 0.0360)  0.338019 | -0.0096  (-0.0268, 0.0075)  0.271719 | 0.0028  (-0.0253, 0.0309)  0.846102 |
|  |  | Group 4 | -0.0118  (-0.0364, 0.0127)  0.344033 | 0.0178  (-0.0242, 0.0598)  0.406502 | -0.0102  (-0.0343, 0.0139)  0.406188 | 0.0174  (-0.0223, 0.0572)  0.390476 | -0.0058  (-0.0332, 0.0215)  0.675145 | -0.0073  (-0.0536, 0.0389)  0.755577 |
| Aged ≥ 60 years | Model 1 | Group 1 | Reference (0) | Reference (0) | Reference (0) | Reference (0) | Reference (0) | Reference (0) |
|  |  | Group 2 | -0.0035  (-0.0276, 0.0206)  0.777697 | -0.0057  (-0.0345, 0.0232)  0.700727 | 0.0020  (-0.0186, 0.0226)  0.848830 | 0.0105  (-0.0140, 0.0350)  0.400359 | -0.0063  (-0.0328, 0.0202)  0.641086 | -0.0081  (-0.0414, 0.0253)  0.635776 |
|  |  | Group 3 | -0.0045  (-0.0321, 0.0231)  0.749487 | 0.0212  (-0.0223, 0.0648)  0.339535 | 0.0060  (-0.0175, 0.0296)  0.616648 | 0.0072  (-0.0298, 0.0442)  0.703520 | -0.0102  (-0.0406, 0.0201)  0.509594 | -0.0017  (-0.0522, 0.0488)  0.947900 |
|  |  | Group 4 | **-0.0478**  **(-0.0842, -0.0114)**  **0.010245** | -0.0325  (-0.0745, 0.0094)  0.128944 | **-0.0368**  **(-0.0679, -0.0058)**  **0.020263** | -0.0295  (-0.0652, 0.0061)  0.105262 | -0.0299  (-0.0699, 0.0101)  0.143098 | -0.0043  (-0.0529, 0.0443)  0.862538 |
|  | Model 2 | Group 1 | Reference (0) | Reference (0) | Reference (0) | Reference (0) | Reference (0) | Reference (0) |
|  |  | Group 2 | -0.0000  (-0.0196, 0.0195)  0.996518 | -0.0199  (-0.0433, 0.0035)  0.096375 | 0.0005  (-0.0174, 0.0184)  0.956021 | -0.0035  (-0.0252, 0.0182)  0.752637 | -0.0008  (-0.0239, 0.0223)  0.945269 | -0.0185  (-0.0482, 0.0113)  0.224561 |
|  |  | Group 3 | -0.0123  (-0.0350, 0.0104)  0.288217 | -0.0164  (-0.0523, 0.0195)  0.371543 | -0.0094  (-0.0303, 0.0114)  0.376169 | -0.0265  (-0.0597, 0.0067)  0.118734 | -0.0079  (-0.0347, 0.0189)  0.563794 | -0.0307  (-0.0764, 0.0150)  0.188002 |
|  |  | Group 4 | **-0.0410**  **(-0.0703, -0.0118)**  **0.006117** | **-0.0465**  **(-0.0803, -0.0127)**  **0.007180** | **-0.0322**  **(-0.0591, -0.0053)**  **0.019061** | **-0.0379**  **(-0.0691, -0.0066)**  **0.017955** | -0.0294  (-0.0640, 0.0052)  0.095970 | -0.0195  (-0.0625, 0.0234)  0.373256 |
|  | Model 3 | Group 1 | Reference (0) | Reference (0) | Reference (0) | Reference (0) | Reference (0) | Reference (0) |
|  |  | Group 2 | 0.0021  (-0.0173, 0.0215)  0.834974 | -0.0185  (-0.0415, 0.0046)  0.116617 | 0.0028  (-0.0152, 0.0207)  0.762579 | -0.0022  (-0.0237, 0.0194)  0.845132 | -0.0007  (-0.0239, 0.0225)  0.954573 | -0.0216  (-0.0515, 0.0084)  0.159052 |
|  |  | Group 3 | -0.0118  (-0.0344, 0.0108)  0.304891 | -0.0034  (-0.0391, 0.0324)  0.853646 | -0.0086  (-0.0295, 0.0123)  0.420010 | -0.0134  (-0.0469, 0.0200)  0.432032 | -0.0084  (-0.0354, 0.0186)  0.543964 | -0.0341  (-0.0806, 0.0124)  0.150767 |
|  |  | Group 4 | **-0.0336**  **(-0.0630, -0.0042)**  **0.025216** | -0.0330  (-0.0666, 0.0006)  0.054686 | -0.0262  (-0.0534, 0.0010)  0.058949 | -0.0270  (-0.0585, 0.0044)  0.092492 | -0.0262  (-0.0613, 0.0090)  0.144611 | -0.0206  (-0.0643, 0.0230)  0.354959 |
| Men | Model 1 | Group 1 | Reference (0) | Reference (0) | Reference (0) | Reference (0) | Reference (0) | Reference (0) |
|  |  | Group 2 | 0.0075  (-0.0094, 0.0244)  0.385178 | 0.0031  (-0.0250, 0.0313)  0.827765 | 0.0144  (-0.0020, 0.0307)  0.084975 | 0.0111  (-0.0153, 0.0376)  0.410213 | 0.0091  (-0.0092, 0.0275)  0.329302 | -0.0073  (-0.0379, 0.0234)  0.641654 |
|  |  | Group 3 | 0.0001  (-0.0204, 0.0206)  0.992738 | 0.0311  (-0.0064, 0.0687)  0.104597 | 0.0114  (-0.0084, 0.0312)  0.259421 | 0.0282  (-0.0070, 0.0635)  0.117076 | 0.0064  (-0.0158, 0.0286)  0.573526 | 0.0069  (-0.0340, 0.0477)  0.742068 |
|  |  | Group 4 | -0.0304  (-0.0624, 0.0016)  0.062611 | -0.0477  (-0.0963, 0.0009)  0.054743 | -0.0271  (-0.0580, 0.0038)  0.085599 | **-0.0462**  **(-0.0918, -0.0006)**  **0.047323** | -0.0332  (-0.0679, 0.0014)  0.060306 | -0.0121  (-0.0649, 0.0408)  0.654392 |
|  | Model 2 | Group 1 | Reference (0) | Reference (0) | Reference (0) | Reference (0) | Reference (0) | Reference (0) |
|  |  | Group 2 | -0.0023  (-0.0176, 0.0129)  0.764684 | -0.0146  (-0.0398, 0.0106)  0.256382 | 0.0023  (-0.0124, 0.0169)  0.761585 | -0.0071  (-0.0305, 0.0164)  0.554943 | 0.0051  (-0.0124, 0.0226)  0.567859 | -0.0195  (-0.0483, 0.0093)  0.184334 |
|  |  | Group 3 | **-0.0238**  **(-0.0426, -0.0050)**  **0.013388** | 0.0021  (-0.0320, 0.0362)  0.903800 | -0.0149  (-0.0330, 0.0031)  0.105300 | -0.0029  (-0.0347, 0.0289)  0.857996 | -0.0125  (-0.0341, 0.0091)  0.256391 | -0.0113  (-0.0503, 0.0277)  0.570700 |
|  |  | Group 4 | **-0.0292**  **(-0.0579, -0.0006)**  **0.045852** | -0.0317  (-0.0752, 0.0118)  0.153485 | -0.0232  (-0.0507, 0.0043)  0.098309 | -0.0263  (-0.0668, 0.0143)  0.204577 | **-0.0428**  **(-0.0758, -0.0099)**  **0.010811** | -0.0179  (-0.0677, 0.0319)  0.480907 |
|  | Model 3 | Group 1 | Reference (0) | Reference (0) | Reference (0) | Reference (0) | Reference (0) | Reference (0) |
|  |  | Group 2 | -0.0012  (-0.0163, 0.0140)  0.880306 | -0.0091  (-0.0338, 0.0156)  0.471064 | 0.0038  (-0.0108, 0.0184)  0.610784 | -0.0010  (-0.0243, 0.0224)  0.934665 | 0.0044  (-0.0130, 0.0219)  0.620233 | -0.0157  (-0.0448, 0.0134)  0.290163 |
|  |  | Group 3 | -0.0163  (-0.0353, 0.0027)  0.092318 | 0.0193  (-0.0146, 0.0532)  0.265500 | -0.0087  (-0.0270, 0.0096)  0.351659 | 0.0111  (-0.0209, 0.0430)  0.497499 | -0.0066  (-0.0284, 0.0153)  0.554901 | -0.0058  (-0.0456, 0.0341)  0.776932 |
|  |  | Group 4 | -0.0222  (-0.0508, 0.0064)  0.128260 | -0.0266  (-0.0700, 0.0168)  0.230504 | -0.0172  (-0.0448, 0.0103)  0.220045 | -0.0226  (-0.0635, 0.0184)  0.280528 | **-0.0403**  **(-0.0732, -0.0074)**  **0.016362** | -0.0173  (-0.0683, 0.0337)  0.506984 |
| Non-postmenopausal women | Model 1 | Group 1 | Reference (0) | Reference (0) | Reference (0) | Reference (0) | Reference (0) | Reference (0) |
|  |  | Group 2 | 0.0243  (-0.0026, 0.0512)  0.077244 | -0.0070  (-0.0432, 0.0292)  0.705236 | 0.0191  (-0.0073, 0.0456)  0.156960 | -0.0012  (-0.0349, 0.0325)  0.943852 | **0.0271**  **(0.0007, 0.0536)**  **0.044944** | -0.0284  (-0.0676, 0.0107)  0.155601 |
|  |  | Group 3 | **0.0436**  **(0.0119, 0.0753)**  **0.007247** | -0.0062  (-0.0574, 0.0450)  0.811806 | **0.0403**  **(0.0091, 0.0715)**  **0.011556** | 0.0018  (-0.0458, 0.0494)  0.941948 | 0.0311  (-0.0001, 0.0623)  0.050935 | 0.0014  (-0.0539, 0.0567)  0.960347 |
|  |  | Group 4 | -0.0129  (-0.0561, 0.0303)  0.557961 | 0.0233  (-0.0431, 0.0897)  0.492506 | -0.0067  (-0.0492, 0.0358)  0.756423 | 0.0323  (-0.0295, 0.0941)  0.306319 | 0.0364  (-0.0061, 0.0790)  0.093750 | -0.0098  (-0.0816, 0.0619)  0.788438 |
|  | Model 2 | Group 1 | Reference (0) | Reference (0) | Reference (0) | Reference (0) | Reference (0) | Reference (0) |
|  |  | Group 2 | 0.0105  (-0.0134, 0.0344)  0.389387 | -0.0019  (-0.0337, 0.0300)  0.909052 | 0.0038  (-0.0201, 0.0276)  0.756342 | 0.0042  (-0.0262, 0.0345)  0.789065 | 0.0214  (-0.0040, 0.0468)  0.099727 | -0.0230  (-0.0595, 0.0135)  0.218466 |
|  |  | Group 3 | 0.0188  (-0.0095, 0.0472)  0.193820 | -0.0206  (-0.0658, 0.0245)  0.371234 | 0.0144  (-0.0139, 0.0426)  0.319679 | -0.0138  (-0.0570, 0.0293)  0.530096 | 0.0141  (-0.0160, 0.0442)  0.359269 | -0.0175  (-0.0693, 0.0343)  0.507772 |
|  |  | Group 4 | -0.0123  (-0.0504, 0.0257)  0.526195 | 0.0130  (-0.0450, 0.0710)  0.660683 | -0.0084  (-0.0464, 0.0295)  0.662848 | 0.0223  (-0.0331, 0.0777)  0.430189 | 0.0313  (-0.0092, 0.0717)  0.130060 | -0.0214  (-0.0880, 0.0451)  0.528984 |
|  | Model 3 | Group 1 | Reference (0) | Reference (0) | Reference (0) | Reference (0) | Reference (0) | Reference (0) |
|  |  | Group 2 | 0.0150  (-0.0088, 0.0387)  0.216983 | -0.0042  (-0.0366, 0.0281)  0.798444 | 0.0071  (-0.0168, 0.0309)  0.562374 | -0.0003  (-0.0317, 0.0312)  0.986806 | 0.0216  (-0.0037, 0.0470)  0.094947 | -0.0349  (-0.0726, 0.0028)  0.070725 |
|  |  | Group 3 | 0.0176  (-0.0107, 0.0459)  0.222719 | -0.0017  (-0.0500, 0.0467)  0.946446 | 0.0105  (-0.0180, 0.0389)  0.471360 | -0.0036 (-0.0506, 0.0434) 0.881629 | 0.0100 (-0.0202, 0.0402) 0.514721 | -0.0096 (-0.0659, 0.0468) 0.739904 |
|  |  | Group 4 | -0.0047  (-0.0428, 0.0334)  0.807687 | 0.0233  (-0.0361, 0.0827)  0.442852 | -0.0031  (-0.0414, 0.0352)  0.873600 | 0.0322  (-0.0256, 0.0899)  0.275794 | 0.0376  (-0.0031, 0.0782)  0.070724 | -0.0171  (-0.0863, 0.0521)  0.629330 |
| Postmenopausal women | Model 1 | Group 1 | Reference (0) | Reference (0) | Reference (0) | Reference (0) | Reference (0) | Reference (0) |
|  |  | Group 2 | -0.0006  (-0.0241, 0.0228)  0.957467 | 0.0103  (-0.0150, 0.0356)  0.425860 | 0.0048  (-0.0171, 0.0268)  0.666622 | 0.0138  (-0.0091, 0.0368)  0.237407 | -0.0040  (-0.0297, 0.0216)  0.757956 | -0.0035  (-0.0329, 0.0259)  0.815929 |
|  |  | Group 3 | 0.0016  (-0.0239, 0.0270)  0.902997 | 0.0268  (-0.0088, 0.0624)  0.141202 | 0.0064  (-0.0174, 0.0302)  0.600019 | 0.0153  (-0.0169, 0.0475)  0.352570 | -0.0147  (-0.0425, 0.0131)  0.301033 | 0.0061  (-0.0353, 0.0475)  0.772988 |
|  |  | Group 4 | **-0.0495**  **(-0.0872, -0.0118)**  **0.010281** | **-0.0442**  **(-0.0882, -0.0003)**  **0.049117** | **-0.0440**  **(-0.0793, -0.0087)**  **0.014643** | -0.0391  (-0.0789, 0.0007)  0.054568 | -0.0232  (-0.0644, 0.0180)  0.269780 | -0.0139  (-0.0650, 0.0372)  0.594765 |
|  | Model 2 | Group 1 | Reference (0) | Reference (0) | Reference (0) | Reference (0) | Reference (0) | Reference (0) |
|  |  | Group 2 | -0.0055  (-0.0257, 0.0148)  0.597711 | -0.0108  (-0.0338, 0.0122)  0.358692 | -0.0013  (-0.0199, 0.0174)  0.894123 | -0.0048  (-0.0261, 0.0164)  0.655714 | -0.0061  (-0.0293, 0.0170)  0.602648 | -0.0181  (-0.0458, 0.0097)  0.203591 |
|  |  | Group 3 | -0.0092  (-0.0313, 0.0128)  0.413272 | -0.0052  (-0.0375, 0.0271)  0.751364 | -0.0065  (-0.0268, 0.0138)  0.527682 | -0.0140  (-0.0438, 0.0159)  0.358941 | -0.0198  (-0.0449, 0.0054)  0.123650 | -0.0163  (-0.0553, 0.0228)  0.415056 |
|  |  | Group 4 | **-0.0426**  **(-0.0754, -0.0098)**  **0.011168** | -0.0375  (-0.0768, 0.0018)  0.061835 | **-0.0319**  **(-0.0621, -0.0017)**  **0.038470** | -0.0321  (-0.0684, 0.0042)  0.083739 | -0.0276  (-0.0650, 0.0098)  0.148371 | -0.0102  (-0.0577, 0.0373)  0.674715 |
|  | Model 3 | Group 1 | Reference (0) | Reference (0) | Reference (0) | Reference (0) | Reference (0) | Reference (0) |
|  |  | Group 2 | 0.0007  (-0.0194, 0.0209)  0.943601 | -0.0083 (-0.0310, 0.0143) 0.470804 | 0.0044  (-0.0142, 0.0231)  0.640916 | -0.0035  (-0.0247, 0.0177)  0.747178 | -0.0024  (-0.0257, 0.0208)  0.836333 | -0.0202  (-0.0484, 0.0080)  0.160465 |
|  |  | Group 3 | -0.0051  (-0.0272, 0.0169)  0.648159 | 0.0067 (-0.0250, 0.0384) 0.680427 | -0.0024  (-0.0228, 0.0179)  0.814118 | -0.0031  (-0.0328, 0.0266)  0.836624 | -0.0193  (-0.0447, 0.0062)  0.137945 | -0.0121  (-0.0516, 0.0274)  0.547962 |
|  |  | Group 4 | -0.0324  (-0.0652, 0.0005)  0.053660 | -0.0256  (-0.0646, 0.0133)  0.197606 | -0.0216  (-0.0519, 0.0087)  0.163198 | -0.0208  (-0.0573, 0.0156)  0.263355 | -0.0238  (-0.0616, 0.0140)  0.217535 | -0.0134  (-0.0619, 0.0352)  0.589619 |

Bold variables indicate P value < 0.05.

Model 1: unadjusted model; Model 2: age, sex/menopause status, race, and BMI were adjusted; Model 3: age, sex/menopause status, race, income level, BMI, smoking status, alcohol drinking status, physical activity level, fractures history, glucocorticoid use, family history of osteoporosis, CCI, calcium intake, and vitamin D intake were adjusted.

Group 1: SD=7-8 h/day; Group 2: SD=6 h/day; Group3: SD<6 h/day; Group 4: SD>8 h/day.

BMD, bone mineral density; BMI, body mass index; CCI, Charlson Comorbidity Index; CI, confidence interval; FN, femoral neck; SD, sleep duration; TF, total femur; TS, total spine; 25(OH)D, 25-hydroxyvitamin D.
